# Gasdermin-D activation by SARS-CoV-2 triggers NET and mediate COVID-19 immunopathology

**DOI:** 10.1186/s13054-022-04062-5

**Published:** 2022-07-07

**Authors:** Camila Meirelles S. Silva, Carlos Wagner S. Wanderley, Flavio Protasio Veras, Augusto Velozo Gonçalves, Mikhael Haruo Fernandes Lima, Juliana Escher Toller-Kawahisa, Giovanni Freitas Gomes, Daniele Carvalho Nascimento, Valter V. Silva Monteiro, Isadora Marques Paiva, Cícero José Luíz Ramos Almeida, Diego Brito Caetité, Juliana Costa Silva, Maria Isabel Fernandes Lopes, Letícia Pastorelli Bonjorno, Marcela Cavichioli Giannini, Natalia Brasil Amaral, Maíra Nilson Benatti, Rodrigo Carvalho Santana, Luis Eduardo Alves Damasceno, Bruna Manuella Souza Silva, Ayda Henriques Schneider, Icaro Maia Santos Castro, Juan Carlo Santos Silva, Amanda Pereira Vasconcelos, Tiago Tomazini Gonçalves, Sabrina Setembre Batah, Tamara Silva Rodrigues, Victor Ferreira Costa, Marjorie Cornejo Pontelli, Ronaldo B. Martins, Timna Varela Martins, Danillo Lucas Alves Espósito, Guilherme Cesar Martelossi Cebinelli, Benedito Antônio Lopes da Fonseca, Luiz Osório Silveira Leiria, Larissa Dias Cunha, Eurico Arruda, Helder I. Nakaia, Alexandre Todorovic Fabro, Rene D. R. Oliveira, Dario S. Zamboni, Paulo Louzada-Junior, Thiago Mattar Cunha, José Carlos Farias Alves-Filho, Fernando Queiroz Cunha

**Affiliations:** 1 Center for Research in Inflammatory Diseases (CRID), Ribeirão Preto Medical School, University of São Paulo, Av. Bandeirantes 3900, Monte Alegre, Ribeirão Preto, São Paulo 14049–900 Brazil; 2 Department of Immunology, Ribeirão Preto Medical School, University of São Paulo, Ribeirão Preto, São Paulo Brazil; 3 Department of Pharmacology, Ribeirão Preto Medical School, University of São Paulo, Ribeirão Preto, São Paulo Brazil; 4 Department of Cell and Molecular Biology, Ribeirão Preto Medical School, University of São Paulo, Ribeirão Preto, São Paulo Brazil; 5 Divisions of Clinical Immunology, Emergency, Infectious Diseases, and Intensive Care Unit, Ribeirão Preto Medical School, University of São Paulo, Ribeirão Preto, São Paulo Brazil; 6 Department of Clinical and Toxicological Analysis, Faculty of Pharmaceutical Sciences, University of São Paulo, São Paulo, Brazil; 7 Department of Pathology and Legal Medicine, Ribeirão Preto Medical School, University of São Paulo, Ribeirão Preto, São Paulo Brazil; 8 Virology Research Center of the Ribeirão Preto Medical School, University of São Paulo, Ribeirão Preto, São Paulo Brazil; 9 Hospital Israelita Albert Einstein, São Paulo, Brazil; 10 Department of Internal Medicine, Ribeirão Preto Medical School, University of São Paulo, Ribeirão Preto, São Paulo Brazil

**Keywords:** Neutrophil, Innate immunity, Organ damage, COVID-19, NETs

## Abstract

**Background:**

The release of neutrophil extracellular traps (NETs) is associated with inflammation, coagulopathy, and organ damage found in severe cases of COVID-19. However, the molecular mechanisms underlying the release of NETs in COVID-19 remain unclear.

**Objectives:**

We aim to investigate the role of the Gasdermin-D (GSDMD) pathway on NETs release and the development of organ damage during COVID-19.

**Methods:**

We performed a single-cell transcriptome analysis in public data of bronchoalveolar lavage. Then, we enrolled 63 hospitalized patients with moderate and severe COVID-19. We analyze in blood and lung tissue samples the expression of GSDMD, presence of NETs, and signaling pathways upstreaming. Furthermore, we analyzed the treatment with disulfiram in a mouse model of SARS-CoV-2 infection.

**Results:**

We found that the SARS-CoV-2 virus directly activates the pore-forming protein GSDMD that triggers NET production and organ damage in COVID-19. Single-cell transcriptome analysis revealed that the expression of GSDMD and inflammasome-related genes were increased in COVID-19 patients. High expression of active GSDMD associated with NETs structures was found in the lung tissue of COVID-19 patients. Furthermore, we showed that activation of GSDMD in neutrophils requires active caspase1/4 and live SARS-CoV-2, which infects neutrophils. In a mouse model of SARS-CoV-2 infection, the treatment with disulfiram inhibited NETs release and reduced organ damage.

**Conclusion:**

These results demonstrated that GSDMD-dependent NETosis plays a critical role in COVID-19 immunopathology and suggests GSDMD as a novel potential target for improving the COVID-19 therapeutic strategy.

**Supplementary Information:**

The online version contains supplementary material available at 10.1186/s13054-022-04062-5.

## Background

NETs are networks composed of extracellular DNA containing histones and cytotoxic enzymes, including myeloperoxidase and elastase, which are released by activated neutrophils as part of their microbicidal arsenal [1]. However, NETs can also induce disseminated vascular coagulation, when released into the circulation, as well as tissue damage when released in the extravascular space [2–5]. In this context, NETs have been described as a key mediator of the pathogenesis of various inflammatory conditions, including COVID-19 [5–8].

We and others demonstrated that patients with severe COVID-19 have an increased number of circulating- and lung-infiltrated neutrophils, which release a large number of NETs [7–9], mediating lung epithelial damage and disseminated intravascular coagulation. However, the molecular mechanisms involved in NET release during SARS-CoV-2-induced response were not addressed.

Recent works showed that Gasdermin-D (GSDMD) is critical to the release of NETs [10, 11]. GSDMD cleavage by inflammatory caspases generates two fragments N and C-terminal. The N-terminal (GSDMD-NT) oligomerizes with plasma and nuclear membranes, forming membranes pores that mediate cell death by NETosis [10–13]. During sepsis, the genetic deletion or pharmacological inhibition of GSDMD with disulfiram prevented the formation of NETs, protecting mice from organ damage development and increasing survival [14]. Disulfiram is a drug that inhibits aldehyde dehydrogenase (ALDH) and is used to treat alcoholism [15]. It was demonstrated that disulfiram can act as a potent inhibitor of GSDMD [16], and an observational study based on clinical records from the national US Veterans Affairs healthcare system revealed a reduced risk of SARS-CoV-2 infection and deaths in individuals treated with disulfiram [17]. Although NETosis is critical for the outcome of COVID-19 [6–8], the mechanisms by which SARS-CoV-2 induced NETs remain unclear.

In the present study, we identified a key role of the GSDMD pathway on NET release during COVID-19. Notably, the treatment with disulfiram abrogated NET formation reducing inflammation and lung tissue damage in a mouse model of COVID-19. These findings indicate GSDMD as a novel potential target for improving the COVID-19 therapeutic strategy.

## Methods

### Analysis of single-cell RNA-seq data

We analyzed single-cell transcriptomic data from bronchoalveolar lavage fluid (BALF) from patients with varying severity of COVID-19 disease and their respective healthy controls from a previously published cohort [18]. The dataset generated by authors is publicly available at https://covid19-balf.cells.ucsc.edu/. The dataset was downloaded, and the RDS file was imported into R environment version v4.0.5. UMAP plots were generated using Seurat v4 [19]. The overlap between gene lists was performed using the UpSetR package [20].

### Patients

The inclusion criteria were individuals hospitalized with moderate or severe forms of COVID-19 diagnosed by RT-PCR in nasopharyngeal swab specimens and lung computed tomography scan involvement compatible with COVID-19 pneumonia; older than 18 years; body weight >50 kg; normal levels of serum Ca^2+^ and K^+^; QT interval normal levels of serum Ca^2+^ and K^+^; QT interval < 450 ms at 12 derivations electrocardiogram (according to the Bazett formula) and negative serum or urinary β-HCG if a woman under 50. The exclusion criteria were defined as diarrhea resulting in dehydration; pregnancy or lactation; metastatic cancer or immunosuppressive chemotherapy and inability to understand the consent form [21]. The moderate form was defined in patients with fever, dyspnea, imaging findings of pneumonia but with SatO_2_>94% on the first day of admission. The severe form was defined in those patients with the same findings of moderate form plus respiratory rate ≥ 30 times per minute or SatO_2_ ≤ 93% and all of them required mechanical ventilation. Both forms were established on the first day of hospitalization [22]. Importantly, the samples were collected on the day of admission. Although the patients were classified as moderate at the time of hospital admission, they received nasal oxygen supplementation during hospitalization, but none required mechanical ventilation. Peripheral blood samples and airway fluid were collected from 63 and 11 confirmed individuals hospitalized with COVID-19, respectively. In the present study, the SARS-CoV-2-negative control group (blood *n*=20, and airway lavage *n*=8) was designed to include matched subjects of older age and chronic non-communicable diseases (age, 40.57 ± 15.29; 24% female). The clinical features of the subjects are detailed in Table 1. The study was approved by the National Ethics Committee, Brazil (CONEP, CAAE: 30248420.9.0000.5440). Written informed consent was obtained from recruited patients. Lung tissue from 10 patients with COVID-19 was autopsied with the minimally invasive ultrasound-guided approach. The autopsy study was approved under protocol number 32475220.5.0000.5440 by HC-FMRP-USP Ethics Committee. Lung tissue samples were used for immunostaining previously described [7, 24]. We used, as non-COVID-19 control, lung samples from the autopsy of two patients with acute myocardial infarction under familial consent.

**Table 1:** Clinical and laboratory characteristics of COVID-19 patients

Severity	Moderate	Severe	*P* value
Demographics and clinical characteristics of COVID-19 patients			
Number	18	45	
Age	63.29 ± 16.58	58.62 ± 16.85	ns
Female	10 (55.55%)	22 (48.88%)	ns
Respiratory status			
Mechanical ventilation	0	45 (100%)	
Nasal-cannula oxygen^Ω^	18 (100%)	0	
Room air	0	0	
Outcome			
Secondary infection	0	12 (26.66%)	0.013
Deaths	6 (33.33%)	13 (28.88%)	ns
Clinical—Comorbidities			
Diabetes mellitus	6 (33.33%)	6 (13.33%)	ns
Cardiopathy	3 (16.66%)	5 (11.11%)	ns
Nephropathy	1 (5.55%)	7 (15.55%	ns
Pneumopathy	4 (22.22%)	6 (13.33%)	ns
Autoimmune diseases	1 (5.55%)	2 (4.44%)	ns
Cancer	2 (11.11%)	2 (4.44%)	ns
Stroke	2 (11.11%)	1 (2.22%)	ns
Obesity	5 (27.77%)	21 (48.88%)	ns
Arterial hypertension	9 (50.0%)	23 (51.11%)	ns
Immunodeficiency	2 (11.11%)	3 (6.66%)	ns
Smoking	5 (27.77%)	9 (20.0%)	ns
Laboratorial Findings			
CRP (mg/dL)^*^	9.90 ± 6.18	12.21 ± 10.13	ns
D-Dimers (μg/mL)^**^	1.48 ± 1.61	3.30 ± 3.55	0.007
LDH (U/L)^#^	428.89 ± 205.23	746.60 ± 429.50	0.002
Ferritin (ηg/mL)^&^	911.01 ± 712.94	1109.47 ± 1547.36	ns
Neutrophils × 10^3^/μL	6.0 ± 4.2	7.5 ± 3.6	0.02
Lymphocytes × 10^3^/μL	1.4 ± 0.8	1.9 ± 1.5	ns
Neutrophil/lymphocytes ratio^¥^	4.2 ± 5.25	3.9 ± 2.4	ns
Platelets × 10^3^/μL	242.7 ± 103.8	287.3 ± 121.1	ns

^**Ω**^The patients were classified as moderate at the time of hospital admission, and they received nasal oxygen supplementation during hospitalization, but none required mechanical ventilation

*ns* not significant

^*^CRP, C-reactive protein (normal value < 0.5 mg/dl);

^**^D-dimers (normal value < 0.5 μg/ml);

^#^LDH, lactic dehydrogenase (normal range: 120–246 U/liter);

^&^Ferritin (normal range, 10–291 ng/ml)

^¥^ > 2.6937 were categorized as the exposed group

### Plasma and neutrophils isolation

Human circulating neutrophils from patients and healthy controls were isolated using Percoll density gradients. Peripheral blood samples were collected by venipuncture and centrifuged at 450 × g for plasma separation. The blood cells were then resuspended in Hank’s balanced salt solution (Corning; cat. 21-022-CV), and the neutrophil population was isolated by Percoll (GE Healthcare; Cat. 17-5445-01) density gradient (72%, 63%, 54%, and 45%). Isolated neutrophils were resuspended in RPMI 1640 (Corning; cat. 15-040-CVR) supplemented with 0.5% BSA. The neutrophil purity was >95% was determined by Rosenfeld-colored Cytospin (Laborclin; cat. 620529).

### Airway lavage

As previously described [7], the airway fluid from 11 patients with COVID-19 patients was obtained by aspiration of the orotracheal tube. This fluid was mixed 1:1 with 0.1 M dithiothreitol (Thermo Fisher Scientific; cat. R0862), incubated for 15 min stirring every 5 min at 37 °C. In the control group (*n*=8), the airway lavage was obtained by injecting sterile isotonic saline solution through a nasal fossa. The injected solution was mixed with nasal and nasopharyngeal secretions before being evacuated from the other nostril when it was collected directly in a sterile tube. The samples were centrifuged at 750 g at 4 °C for 10 min. The supernatants were used for measurement of NETs, and the cells were fixed for immunostaining in coverslips with Poly-L-lysine solution 0.1% (Sigma-Aldrich; cat P8920).

### NETs quantification by MPO/DNA conjugates in picogreen assay

This procedure was performed as previously described [4]. Briefly, an antibody bound to a 96-well clear-bottom black plate captured the enzyme MPO (5 μg/ml; Abcam). Neutrophils (10^6^ cells) obtained from COVID-19 severe patients or healthy controls in RPMI 1640 supplemented with 0.5% BSA were treated or not. According to the manufacturer's instructions, the amount of DNA bound to the enzyme was quantified using the Quant-iT™ PicoGreen® kit (Invitrogen). The fluorescence intensity (excitation at 488 nm and emission at 525 nm) was quantified by a FlexStation 3 Microplate Reader (Molecular Devices, CA, USA). The concentration of NETs in supernatants was determined, and 4 h after infection, NETs were determined in supernatants. In another context, neutrophils from healthy controls were treated or not 1 h before infection with SARS-CoV-2 (MOI = 1.0), *Staphylococcus aureus* obtained from American Type Culture Collection (ATCC, USA) number 6538 (3:1), or *Streptococcus pneumoniae* (TIGR4) (1:1) and incubated for 4 h at 37 °C. Next, NETs amounts were analyzed by picogreen assay. Furthermore, plasma or supernatants of airway fluid were incubated for 4 h at 37 °C for determination of NETs.

### Human GSDMD ELISA Kit

GSDMD *in vitro* SimpleStep ELISA® (Enzyme-Linked Immunosorbent Assay) kit (ab272463) was used for the quantitative measurement of GSDMD in human plasma from COVID-19 moderate or severe and healthy control samples according to the manufacturer’s instructions. To perform the assay, samples or standards were added to the wells, followed by the antibody mix. TMB Development Solution was added, and this reaction was then stopped by the addition of Stop Solution. The signal was measured at 450 nm.

### Immunofluorescence assay and confocal microscopy

Human and mice lung sections or isolated neutrophils were fixed with paraformaldehyde (4%). The samples were washed with PBS and blocked with 1% BSA, 22.5 mg/mL glycine in PBST (PBS + 0.1% Tween 20). The slides were overnight stained with the following antibodies: a) Mouse anti-myeloperoxidase (MPO, 2C7, Abcam, cat. ab25989, 1:500); B) Rabbit anti-GSDMD-NT (Abcam, cat. ab215203). Next, the samples were incubated with alpaca anti-mouse IgG Alexa Fluor 488 (Jackson ImmunoResearch, cat. 615-545-214, 1:1000) or alpaca anti-rabbit IgG Alexa Fluor 594 (Jackson ImmunoResearch, cat. 611-585-215, 1:1000) secondary antibodies for 30 minutes. The nuclei were stained with 4',6-diamidino- 2-phenylindole, dihydrochloride (DAPI, Life Technologies, cat. D1306, 1:1.000). The Axio Observer acquired images combined with the LSM 780 confocal microscope system (Carl Zeiss) at a 630× magnification. All images acquired were analyzed using Fiji by ImageJ. Finally, 10 fields per sample were randomically analyzed at the x and y focal plane.

### Cells, virus, and mock

To obtain the SARS-CoV-2 virus or the control (Mock), the SARS-CoV-2 Brazil/SPBR- 02/2020 strain was isolated from a nasopharyngeal swab of the first confirmed Brazilian cases of COVID-19 and expanded on Vero-E6 (African green monkey kidney) cells. Vero-E6 cells were cultured in DMEM high glucose supplemented with 10% fetal bovine serum (FBS; HyClone, Logan, Utah), 100 U/mL penicillin, 1% μg/mL streptomycin (P/S Corning; cat. 30-002-CI), 1% glutamine (Corning; cat. 15718008), 1% hepes (Corning; cat. 25-060-CI), and 1% fungizone (Gibco; cat. 15290-018) at 37°C in the 5% CO_2_ atmosphere. Experiments were performed after one passage in cell culture when Vero-E6 cells with DMEM plus 2% FBS in 150 cm^2^ surface area flasks were incubated at 37 °C in a 5% CO_2_ atmosphere. All procedures related to virus culture were handled at a biosafety level 3 (BSL3) multi-user facility, according to WHO guidelines. Virus titers were determined as the tissue culture infectious dose at 50% (TCID 50/mL). Virus stocks were kept in −80 °C ultra-freezers. The virus strain was sequenced to confirm the virus identity and its complete genome is publicly deposited (GenBank accession No. MT710714). Non-infected control cultures (mock) were prepared using pure non-supplemented DMEM as inoculum.

### Mouse infection and treatment

To evaluate the effects of Disulfiram *in vivo*, we infected the K18-hACE2 humanized mice (B6.Cg-Tg(K18-ACE2)2Prlmn/J) (McCray et al. [25] Oladunni et al. [26], Bao et al. [27]). K18-hACE2 mice were obtained from The Jackson Laboratory and were bred in the Centro de Criação de Animais Especiais (Ribeirão Preto Medical School/University of São Paulo). For the experimental infection, animals were transferred to the BSL3 facility. Eight-week-old male K18-hACE2 mice were infected with 2 × 10^4^ PFU of SARS-CoV-2 (in 40 µL) by the intranasal route as previously described [26]. Twenty-four hours after the virus inoculation and once daily until day 5 post-infection (dpi), animals were treated with Disulfiram (50 mg/kg, i.p.) (*n* = 7) or vehicle (*n* = 7). Naive mice remained uninfected and untreated. On the 5 dpi, 6 h after the injection of Disulfiram or vehicle, animals were humanely euthanized for samples collection. All the experimental procedures were performed in accordance with the guide for the use of laboratory animals of the University of Sao Paulo and approved by the institutional ethics committee (066/2020).

### Drugs

To assess the pathways involved in the release of NETs, neutrophils were treated with GSDMD inhibitor—Disulfiram—(Sanofi-Aventis South America)—(3, 10, or 30 μM); RNA polymerase inhibitor—TDF—(tenofovir disoproxil fumarate)—CAS 202138-50-9—(10 μM); neutralizing anti-hACE2 antibody-αACE2-Rhea Biotech; Cat. IM-0060—(0.5 μg/ml); serine protease TMPRSS2—camostat mesylate—Cat. SML0057—(10 μM); pan-caspase inhibitor-Z-VAD-FMK-CAS 187389-52-2—(50 μM); caspase-1 Inhibitor I-CAS 143313-51-3—(25 μM); or vehicle (DMSO, <1% v/v) 1 h before stimulation.

### Plaque reduction neutralization test using disulfiram against SARS-CoV-2

A PRNT (Plaque Reduction Neutralization Test) was performed in VERO E6 cells on a cell-monolayer in 24-well plates to evaluate the effect of disulfiram on SARS-CoV-2 infection. A serial dilution of disulfiram was made in MEM medium, no FBS, using a 4-fold dilution factor, from 1mM to 0.0156 mM and then incubated 1 hour at 37 ºC with approximately 90 PFU (Plaque Forming Units) of SARS-CoV-2. Cells were washed twice with a saline buffer (PBS), and the complex virus+disulfiram was added to the confluent monolayer. Plates were incubated for one hour at 37 ºC to allow virus adsorption. Cells were washed with PBS twice, and a semi-solid carboxymethyl cellulose (CMC) medium (1.5% in DMEM) was added to the cells and incubated four days at 37 ºC, 5% CO_2_. The overlay was removed, and cells were fixed with formalin 10% buffer and then stained for 15 minutes in 1% crystal violet. PRNT50 value was calculated using a curve fitting method (Nonlinear dose-response regression) to a more precise result. The data are expressed as relative PFU (%) to that for the untreated virus-infected control cells, which was defined as 100%.

### Cytopathic effect of SARS-CoV-2 infected neutrophils on A549 and HUVEC cell lines by flow cytometry

Neutrophils were isolated from healthy controls (1 × 10^6^) incubated or not with disulfiram (10 μM) for 1 h, and, next, were incubated or not with SARS-CoV-2 (MOI = 1.0) for 1 h. Then, the infected neutrophils were washed twice and co-cultured with Human alveolar basal epithelial (A549) or Human Umbilical Vein Endothelial Cells (Huvec) cell lines (2 × 10^5^) previously stained with CellTrace™ Violet Cell Proliferation Kit (Thermo Fisher #C34557) following manufacturer protocols, for 24 h at 37 °C. Subsequently, the cells were harvested and suspended in buffer containing 2% FBS in PBS for further evaluation of cell viability by flow cytometry through staining with Fixable and viability dye eFluor™ 780 (eBioscience™) following manufacturer protocol [7]. The acquisition of the cells was performed in a flow cytometer (FACS Verse), and the analyses were made using the FlowJo software (FlowCytometry Analysis Software v10).

### Cytokine assays (ELISA)

The quantification of cytokines in mouse lungs was conducted by a commercial ELISA kit (R&D Systems) according to the manufacturer’s instructions. The individual sample's optical density was measured at 450 nm using a spectrophotometer (Spectra Max-250, Molecular Devices, Sunnyvale, CA, USA).

### Western blot

Neutrophils (3 × 10^6^) from blood were isolated by percoll density gradients as previously described [3] and were lysed with a boiling Laemmli buffer. Samples were loaded onto a 15% SDS-PAGE gel. Proteins were then transferred onto nitrocellulose membranes using Bio-Rad’s Trans-Blot Turbo, which were blocked using 5% nonfat dry milk in 1X TBS-T buffer for 1 h at room temperature. The membranes were incubated overnight at 4 °C under mild agitation in 5% nonfat dry milk in 1X TBS-T buffer containing the following primary antibodies: mouse anti-Caspase-1 (p20) (mAb (Bally-1); Adipogen; AG-20B-0048-C100; 1:1000), mouse anti-Caspase-4 (MBL cat. M029-3; 1:1000), rabbit anti-GSDMD (Abcam, cat. ab215203; 1:1000), rabbit anti-α-actin (Sigma-Aldrich, cat. A2066; 1:5000), mouse anti-α-actin (Cell Signaling, cat. 3700; 1:1000), rabbit anti-RIG-I (D14G6) (Cell Signaling, cat. 3743S; 1:1000). Membranes were washed in 1x TBS-T and incubated with appropriate secondary HRP-conjugated antibodies diluted in 5% nonfat dry milk in 1X TBS-T buffer. Protein detection was done using an ECL™ Prime Western Blotting System (GE Healthcare) and an Amersham Imager 600 (GE Healthcare).

### Analysis of SARS-CoV-2 viral load

The SARS-CoV-2 viral load was determined using the primer-probe sets for 2019-nCoV_N1 and N2 (Integrated DNA Technologies), as previously described by Veras et al. [7]. All RT-PCR assays were done using the Viia 7 Real-time PCR System (Applied Biosystems). The data were represented by fold change relative expression of SARS-CoV-2 group.

### Histological examination

Mice were euthanized 5 days post-SARS-CoV-2 infection. The lung tissue was harvested and fixed in 4% buffered formalin and posteriorly embedded in paraffin blocks. Sections (3 μm) were then stained with hematoxylin and eosin for histological examination. Images were acquired by DMI 6000B microscope (Leica Microsystems) at a 400x magnification. Histological evaluation was performed by a pathologist.

### Statistics

Statistical significance was determined by either two-tailed paired or unpaired Student t test or one-way ANOVA followed by Tukey’s post hoc test. Absolute numbers and percentages were compared with Fisher’s exact test. Spearman’s rank-order correlation (r) was calculated to describe correlations. *P*<0.05 was considered statistically significant. Statistical analyses and graph plots were performed with GraphPad Prism 8.4.2 software.

## Results

### Expression of activated GSDMD is associated with NETosis during COVID-19

**Fig. 1:**
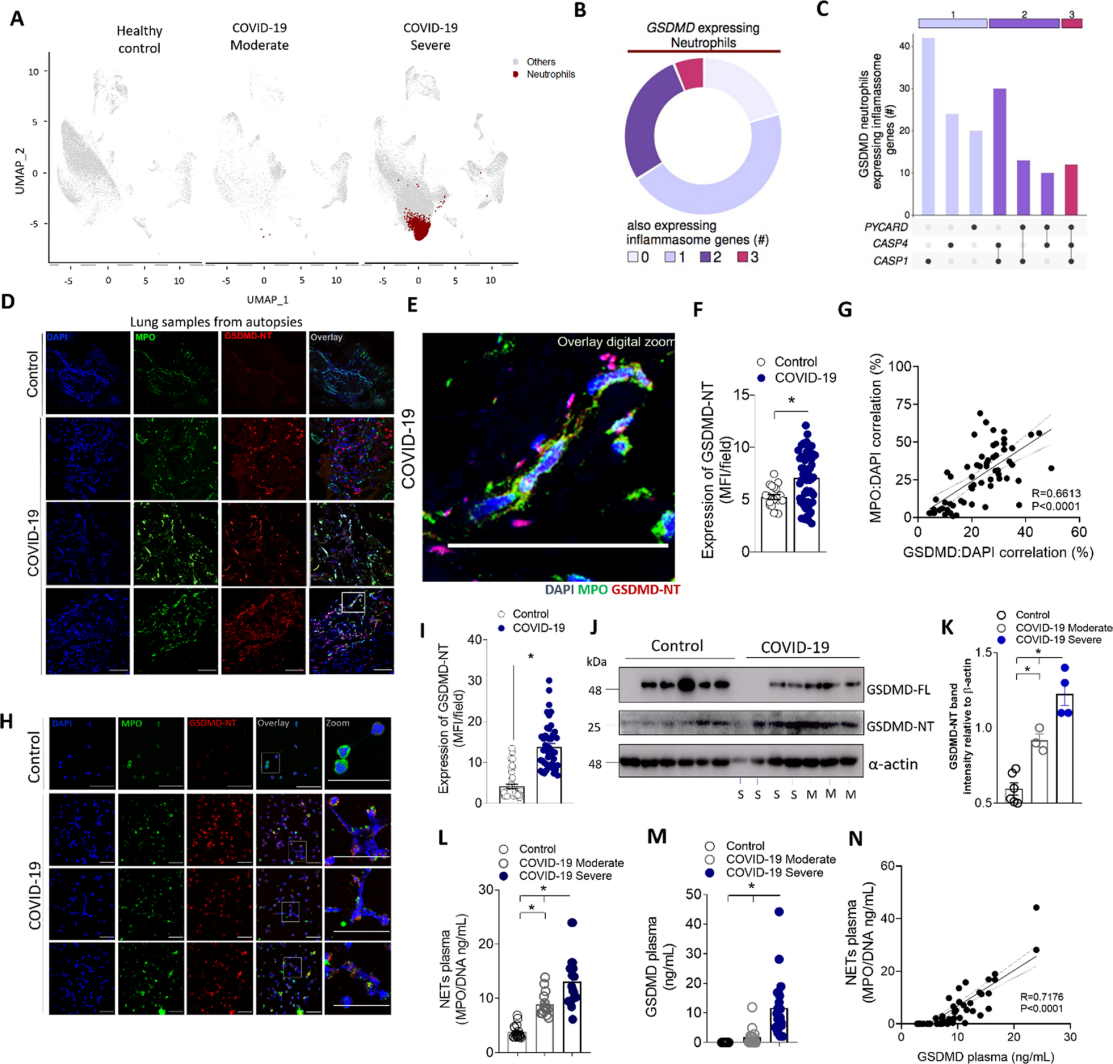
Neutrophils from COVID-19 patients undergoing NETosis express GSDMD. **A** Single-cell analysis of BALF from patients with COVID-19 across severity status (Healthy control, Moderate, and Severe). UMAP visualization from gene expression data of 66,452 cells, highlighting neutrophil expression cluster in severe COVID-19 patients (red) from bronchoalveolar lavage fluid (BALF) cells. **B** Pie chart plot representing the proportion of GSDMD expressing neutrophils. **C** UpSet plot showing the intersection of inflammasome genes expressed in neutrophils, including PYCARD, CASP4, and CASP1, derived from COVID-19 severe patients. The point diagram indicates the intersection among the genes and the bar graph shows the number of GSDMD expressing neutrophils in each intersection (y-axis). **D** Representative confocal analysis of GSDMD-NT and NETs in the lung tissue sample from autopsies of COVID-19 patients (*n* = 6 or control *n* = 3). Immunostaining for DNA (DAPI, blue), myeloperoxidase (MPO, green), and the GSDMD cleaved fraction (GSDMD-NT, red) are shown. The scale bar indicates 50 μm at 630× magnification. **E** Zoomed images of Fig. 1D inset white square. **F** The expression of GSDMD-NT was quantified by MFI per field. **G** Correlation between colocalization GSDMD-NT:DAPI and NETs (MPO:DAPI). **H** Representative confocal analysis of GSDMD and NETs in the blood neutrophils isolated from COVID-19 patients (*n* = 5) or controls (*n* = 5). Cells were stained for DNA (DAPI, blue), MPO (green), and GSDMD-NT (red). Scale bar indicates 50 μm, 4× digital zoom was performed in the inset white square. **I** Expression of GSDMD-NT was quantified by MFI per field. **J** Expression of full-length GSDMD (GSDMD-FL) and active GSDMD (GSDMD-NT) by Western blot. Moderate COVID-19 (M, *n* = 3) severe COVID-19 (S, *n* = 4), and healthy controls (*n* = 36). **(K)** Western blot quantification by densitometry. GSDMD-NT values obtained were normalized to total beta-actin **(L)** Circulating amounts of MPO/DNA-NETs and **M** GSDMD from plasma of patients with moderate COVID-19 (*n* = 15) severe COVID-19 (*n* = 21), and healthy controls (*n* = 320). **N** Correlation between plasmatic levels of MPO/DNA-NETs and GSDMD. The data are expressed as mean ± SEM (*p < 0.05; t test in **F** and **I**, Pearson’s correlation in G and M, one-way ANOVA followed by Tukey’s in **K** and **L**)

To investigate the molecular pathway involved in NETs production in COVID-19, we first performed a single-cell RNA sequencing (scRNA-seq) analysis in public data of bronchoalveolar lavage fluid (BALF) from COVID-19 patients and healthy controls [18]. Confirming previous findings clustering analysis showed that BALFs of patients with severe COVID-19 contained higher proportions of neutrophils than healthy controls or patients with moderate COVID-19 (Fig. 1A). The analysis of gene expression in these neutrophils revealed that *GSDMD* mRNA is expressed in 11.7% and these cells also expressed other inflammasome-related genes (Fig. 1B). We identified neutrophils with three molecular profiles according to the expression of inflammasome genes (*Pycard*, *Casp4,* and *Casp1*) as indicated by linked points (Fig. 1C). Based on these findings and to add more information concerning the role of GSDMD, we analyze the GSDMD protein expression and its activated cleaved fraction (GSDMD-NT). Then, we enrolled 63 hospitalized patients with moderate [*n*=18, age 63 (±16.58) years, and 55.5% women] and severe [*n*=45, age 58 (±16.85) years, and 48.8% women] COVID-19. Assisted mechanical ventilation was implemented in 100% of patients with severe COVID-19. The serum levels of CRP, LDH, and fibrin degradation products D-dimers were found above the normal range, indicating ongoing inflammation, tissue lesions, thrombosis, and subsequent fibrinolysis. The identified causes of death were pneumonia, ARDS, or multi-organ failure. Existing comorbidities are also reported in Table 1. Confirming previous findings [7], COVID-19 patients showed higher levels of NETs in the airway fluid (Fig. S1A). Notably, the cleaved fraction of GSDMD (GSDMD-NT) was found on these NETs (Additional file 1: Fig. S1B). Moreover, the image analysis of lung autopsies from patients who died by COVID-19 showed the presence of NET structure associated with activated GSDMD-NT fraction (Fig. 1D–F). As a control, NETs and GSDMD-NT were not found in the tissues obtained in lung autopsies from patients who died of acute myocardial infarction (Fig. 1D). Furthermore, in lung tissue, it was observed a positive correlation between GSDMD-NT:DAPI colocalization with NETs (MPO:DAPI), confirmed by Pearson’s correlation coefficient analysis (Fig. 1G). Then, we analyzed the expression of GSDMD in blood neutrophils from COVID-19 patients and healthy volunteers. Using confocal microscopy, we found elevated expression of GSDMD accumulated on the neutrophil plasmatic membrane and in typical NETs structures containing extracellular DNA (Fig. 1H and I). The western blot analysis confirmed that neutrophils from severe COVID-19 patients expressed higher levels of GSDMD-NT when compared to moderate and health controls (Fig. 1J–K). Additionally, we observed that patients with severe COVID-19 showed elevated serum levels of NETs and GSDMD than moderate COVID-19 patients and health controls (Fig. 1L and M). A positive correlation was found between serum levels of NETs and GSDMD (Fig. 1N). Thus, these results indicate that the GSDMD pathway could be involved in the process of COVID-19-induced NETosis.

### GSDMD is required for NETs release by neutrophils from COVID-19 patients

**Fig. 2:**
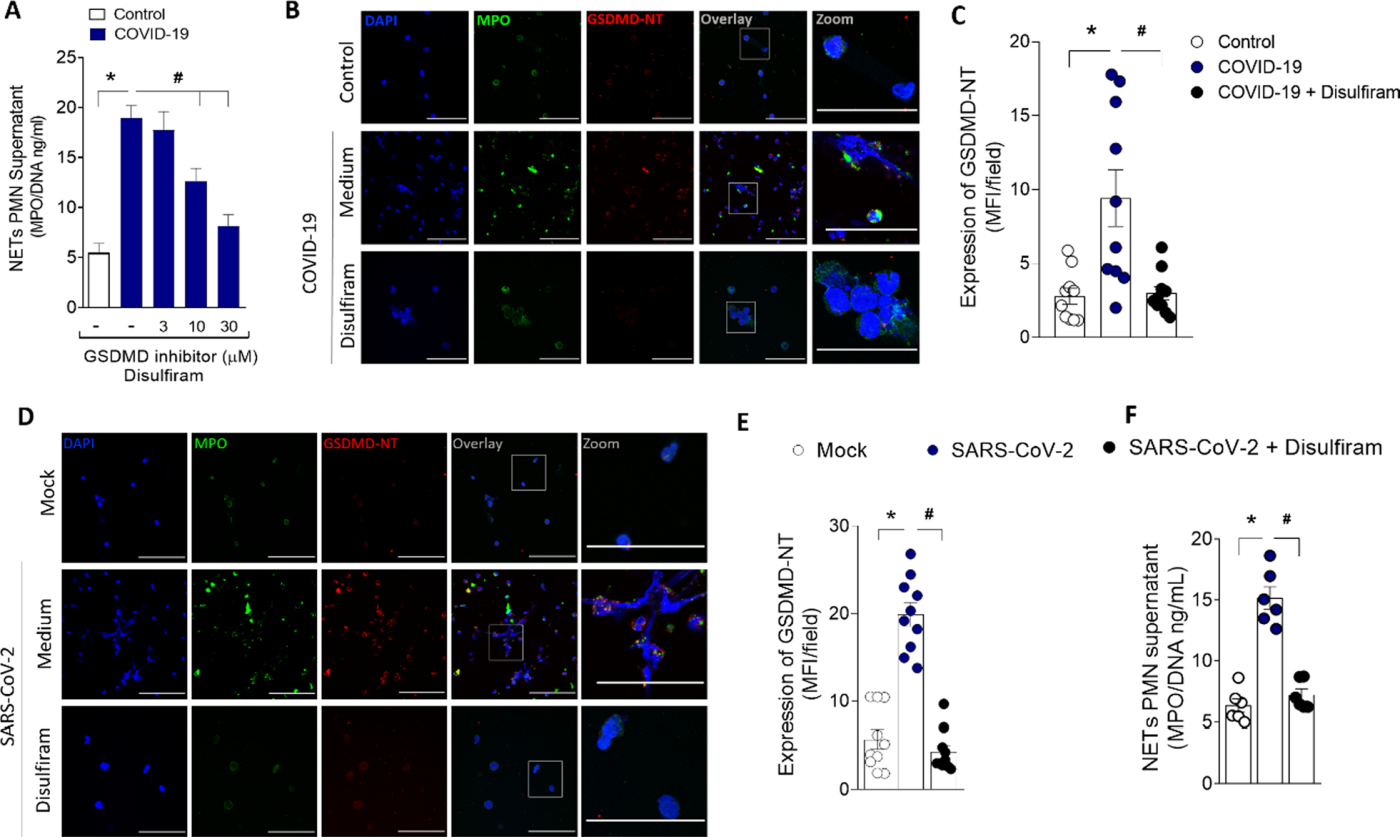
GSDMD activation during COVID-19 mediates NETs formation. Human neutrophils were isolated from healthy control (*n* = 12) and COVID-19 (*n* = 15) patients. Cells were treated with disulfiram (3, 10, and 30 µM) and cultured for 4 h at 37 °C. **A** The concentrations of MPO/DNA-NETs in the supernatants were determined using the picogreen test. **B** Representative immunostaining images for DNA (DAPI, blue), myeloperoxidase (MPO, green), and the GSDMD cleaved fraction (GSDMD-NT, red) are shown. The scale bar indicates 50 μm at 630× magnification. 4 × digital zoom was performed in the inset white square.** C** GSDMD-NT expression was quantified by MFI per field. **D** Human neutrophils were isolated from healthy control (*n* = 6). Cells were treated with disulfiram (30 uM). After 1 h, the cells were incubated with SARS-CoV-2 or Mock (virus control) and cultured for 4 h at 37 °C. Representative immunostaining images for DNA (DAPI, blue), myeloperoxidase (MPO, green), and the GSDMD cleaved fraction (GSDMD-NT, red) are shown. The scale bar indicates 50 μm at 630× fication. 4 × digital zoom was performed in the inset white square. **(E)** GSDMD-NT expression was quantified by MFI per field. **F** The concentrations of MPO/DNA-NETs in the supernatants were determined using the picogreen test. The data are expressed as mean ± SEM (*or ^#^ *p* < 0.05; one-way ANOVA followed by Tukey’s test in **A**, **C**, **E**, and **F**)

Recent studies showed that disulfiram potently inhibits GSDMD [16]. To investigate the potential role of GSDMD on NETs release during COVID-19, we added disulfiram on cell cultures of neutrophils from COVID-19 patients. We found that disulfiram inhibited the release of NETs in a concentration-dependent manner and the expression GSDMD-NT (Fig. 2A–C). Importantly, the treatment with disulfiram also abrogated the activation of GSDMD and the release of NETs by SARS-CoV-2 infection in neutrophils (Fig. 2D–F). Of note, disulfiram, at the used concentrations, did not inhibit the viral replication (Additional file 1: Fig. S2). Moreover, we observed that disulfiram also blocked the NETs production induced by bacteria *Streptococcus pneumoniae* or *Staphylococcus aureus,* known inductors of GSDMD activation (Additional file 1: Fig. S3). These results suggest that GSDMD is involved in the release of NETs triggered by SARS-CoV-2 and also by other microbes.

### The cleavage of GSDMD depends on neutrophil infection and replication by SARS-CoV-2

**Fig. 3:**
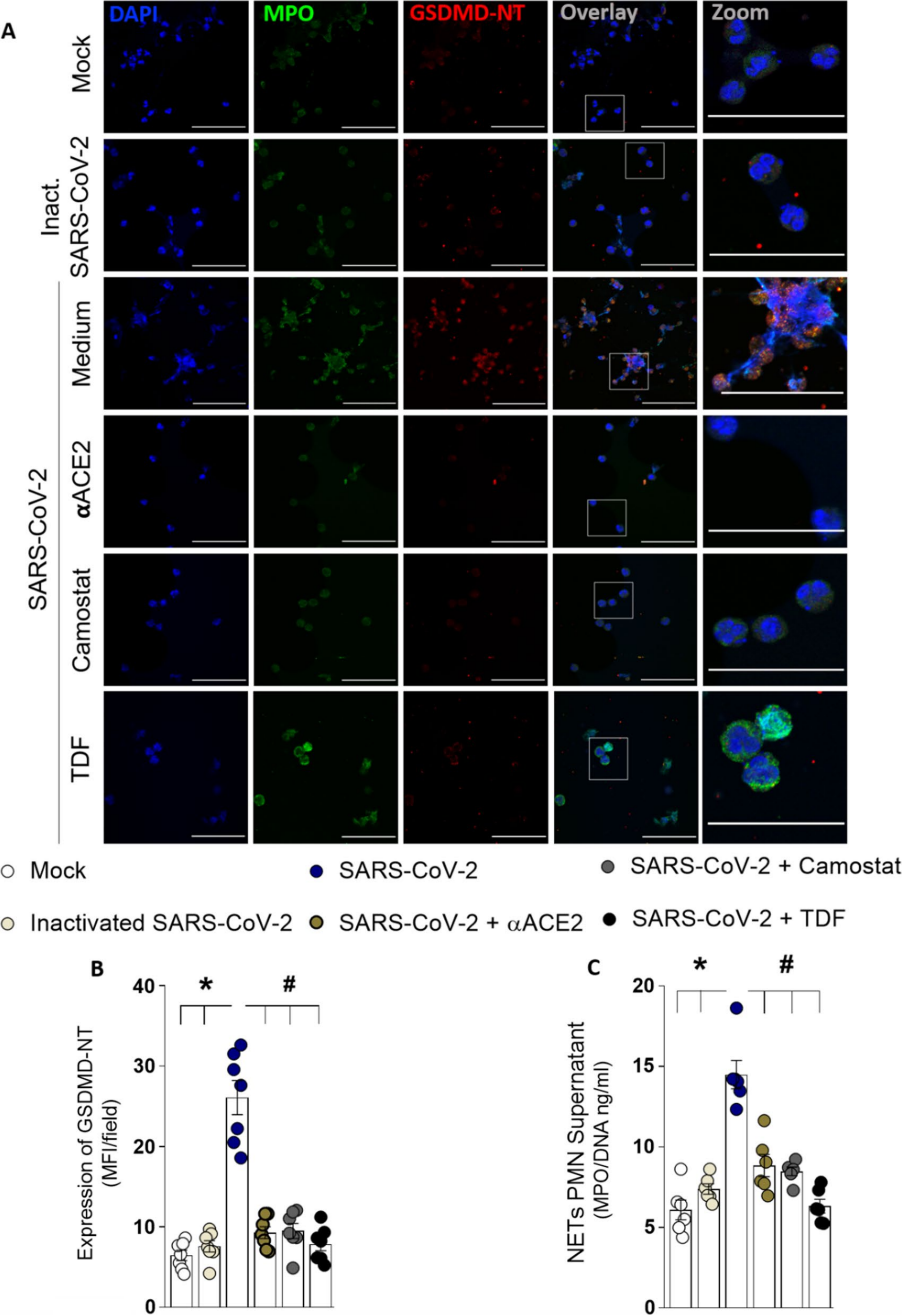
The GSDMD-dependent NETosis is triggered by SARS-CoV-2 directly. Human neutrophils were isolated from healthy control (*n* = 7). Cells were treated with a neutralizing anti-hACE2 antibody (αACE2, 0.5 µg/ml), an inhibitor of the serine protease TMPRSS2 (camostat, 10 µM), or an antiretroviral that reduces SARS-CoV-2 replication through the inhibition of RNA polymerase—tenofovir disoproxil fumarate (TDF; 10 µM). After 1 h, the cells were incubated with SARS-CoV-2, or virus control (inactivated SARS-CoV-2 or Mock) and cultured for 4 h at 37 °C. **A** Representative immunostaining images for DNA (DAPI, blue), myeloperoxidase (MPO, green), and the GSDMD cleaved fraction (GSDMD-NT, red) are shown. The scale bar indicates 50 μm at 630 × magnification. 4× digital zoom was performed in the inset white square. **B** GSDMD-NT expression was quantified by MFI per field. **C** The concentrations of MPO/DNA-NETs in the supernatants were determined using the picogreen test. The data are expressed as mean ± SEM (*or ^#^ *p* < 0.05, one-way ANOVA followed by Tukey’s test in **B** and **C**)

We previously demonstrated that SARS-CoV-2 directly induces NET release by human neutrophils [7]. Thus, considering that SARS-CoV-2 employs ACE2 and TMPRSS2 for host cell entry [28], we investigated whether GSDMD activation in neutrophils is induced by SARS-CoV-2 via ACE2–TMPRSS2 axis during the NETosis process. Neutrophils incubation with the inactivated SARS-CoV- 2 show neither GSDMD activation nor NETs release. Moreover, treatment of isolated neutrophils with a neutralizing anti-hACE2 antibody (αACE2) or camostat, an inhibitor of TMPRSS2, abrogated SARS-CoV-2-induced GSDMD activation and NETs release. Then, we treated SARS-CoV-2 infected neutrophils with tenofovir disoproxil fumarate (TDF), an antiretroviral that reduces SARS-CoV-2 replication through the inhibition of RNA polymerase [29]. Remarkably, TDF also reduced the GSDMD cleavage and release of NETs by neutrophils incubated with SARS-CoV-2 (Fig. 3A–C). Moreover, we confirmed in our experimental conditions that during 4 h, the SARS-CoV-2 is able to infect and replicate in the neutrophils (Additional file 1: Fig. S4). Notably, the treatment with the GSDMD inhibitor, disulfiram, did not affect the viral replication (Additional file 1: Fig. S5). These results suggest that disulfiram inhibits NET production induced by SARS-CoV-2 through GSDMD inhibition, without impact on upstream steps, like viral infection and replication.

### Inflammasome/GSDMD pathway is highly expressed in blood neutrophils from COVID-19 patients

**Fig. 4:**
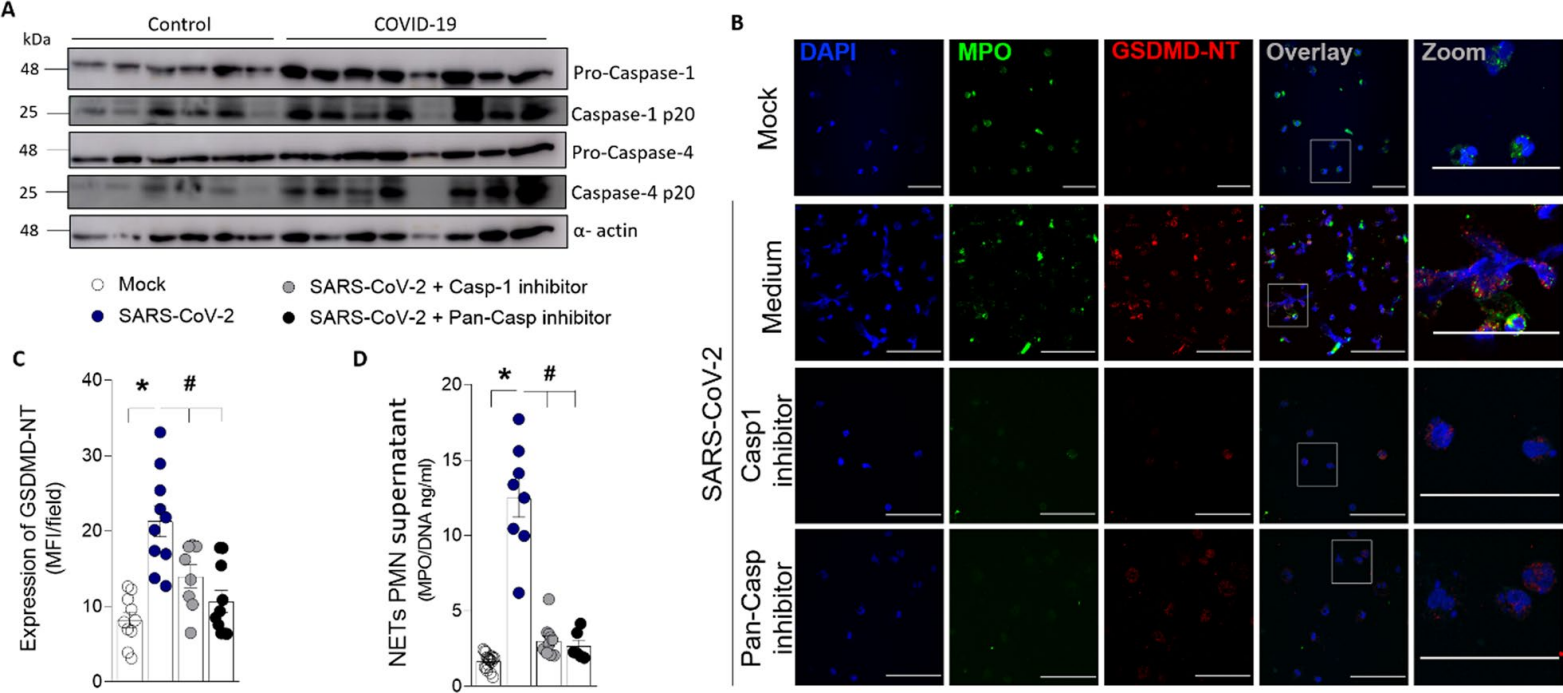
Inflammatory caspases mediate GSDMD cleavage and NETs formation after SARS-Cov-2 neutrophil infection**.** Neutrophils were isolated from healthy controls (*n* = 6) and COVID-19 patients (*n* = 8). **A** The neutrophil lysates were harvested for immunoblot analysis of pro-caspase-1, pro-caspase- 4, and their cleaved fraction caspase-1-p20 and caspase-4-p20. The α-actin was used as a loading control. **B** Human neutrophils were isolated from healthy controls (*n* = 8). Cells were treated with caspase-1 inhibitor (Ac-YVAD-CHO, 25uM) or pan-caspase inhibitor (Z-VAD-FMK, 50uM). After 1 h, the cells were incubated with SARS-CoV-2 or Mock (virus control) and cultured for 4 h at 37 °C. Representative immunostaining images for DNA (DAPI, blue), myeloperoxidase (MPO, green), and the GSDMD cleaved fraction (GSDMD-NT, red) are shown. The scale bar indicates 50 μm at 630× magnification. 4 × digital zoom was performed in the inset white square. **C** GSDMD-NT expression was quantified by MFI per field. **D** The concentrations of MPO/DNA-NETs in the supernatants were determined using the picogreen test. The data are expressed as mean ± SEM (*or ^#^ *p* < 0.05, one-way ANOVA followed by Tukey’s test in **C** and **D**)

It is well established that inflammasome activation by canonical (caspase-1), or non-canonical pathway (caspase-4) cleaves and activates GSDMD [11, 30]. Considering these findings, we investigated whether these pathways are involved in GSDMD activation in COVID-19 patients. Confirming single-cell transcriptome data (Fig 1C), neutrophils from COVID-19 patients also showed increased expression of active caspases-1 and caspase-4 (Fig. 4A). Also, we observed that GSDMD activation and NETs production were abrogated in SARS-CoV-2-infected neutrophils from healthy individuals after treatment with inhibitors of caspase-1 (Ac-YVAD-CHO) or pan-caspases (Z-VAD-FMK) (Fig. 4B–D). Neutrophils from COVID-19 patients also showed increased expression of RIG-I (Additional file 1: Fig. S6), a viral sensor involved in the recognized RNA virus, which is implicated in inflammasome activation [31]. These results indicate that the inflammasome is involved with the cleavage of GSDMD and NETs release triggered by SARS-CoV-2.

### Epithelial and endothelial cell death elicited by SARS-CoV-2–induced NETs requires GSDMD

**Fig. 5:**
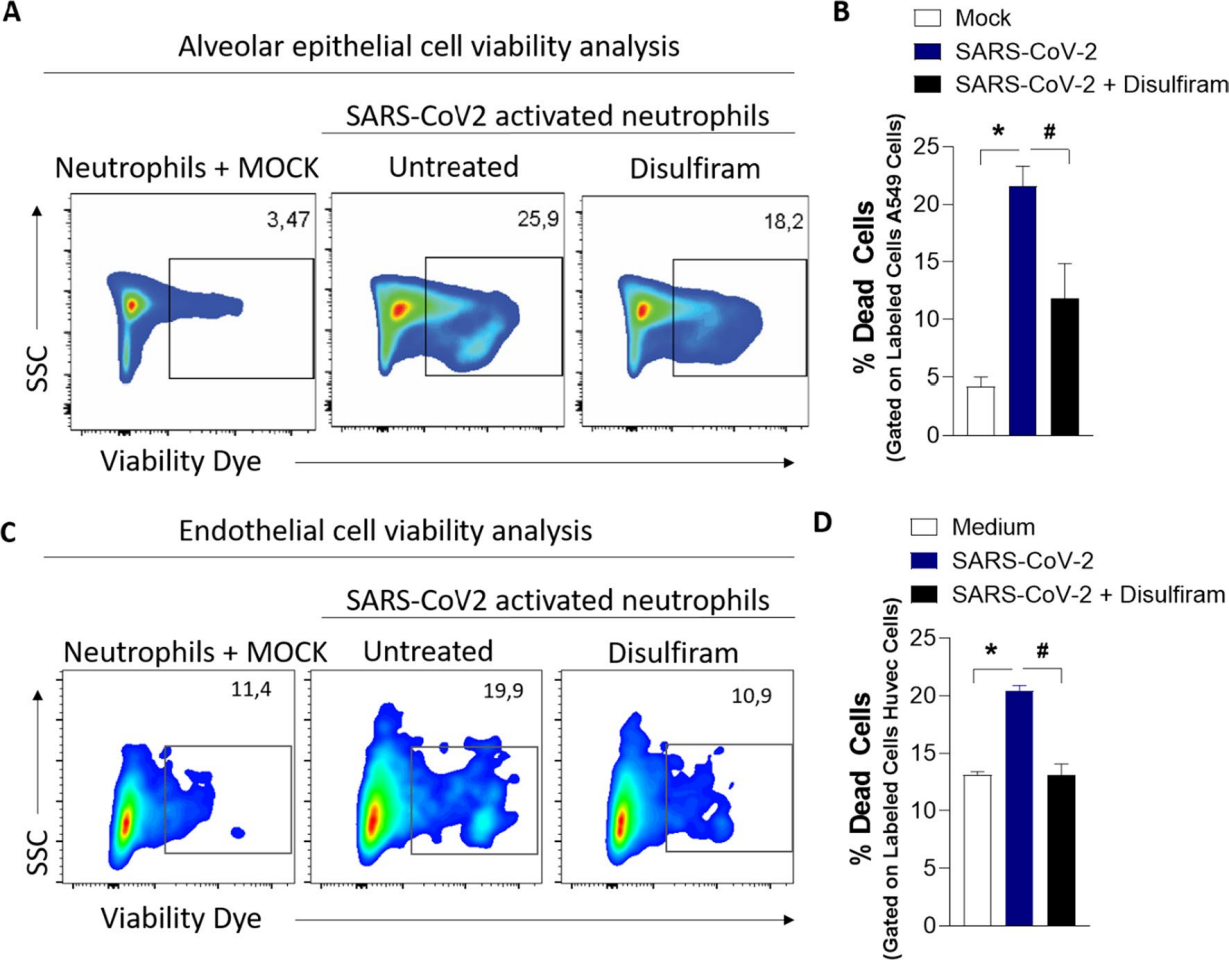
GSDMD inhibition prevents cell damage induced by NETs associated with SARS-CoV-2 infection. Blood isolated neutrophils (1 × 10^6^ cells) from healthy donors, pretreated, or not, with disulfiram (30 µM) were incubated, or not, with SARS-CoV-2 (*n* = 36). After 1 h, these neutrophils were washed twice and co-cultured with lung epithelial cells (A549, 2 × 10^5^ cells) or endothelial cells (HUVEC, 2 × 10^5^ cells) previously stained with viability dye for 24 h at 37 °C. **A** Representative dot plots of FACS analysis for viability dye + A549 cells. **B** Frequency of viability dye + A549 cells. **C** Representative dot plots of FACS analysis of viability dye + HUVEC. **E** Frequency of viability dye + HUVEC cells. Data are representative of at least two independent experiments and are shown as mean ± SEM (*or ^#^ *p* < 0.05, one-way ANOVA followed by Tukey’s test in **B** and **D**)

In several pathological conditions, the release of NETs is associated with tissue damage [2–5, 7]. COVID-19 is characterized by extensive tissue damage, mainly in the lung [7, 9, 32]. Therefore, we investigate whether inhibition of GSDMD prevents NET-induced cell damage. To this end, neutrophils from the blood of healthy controls were treated with disulfiram incubated with SARS-CoV-2 for 1 h; the cells were washed twice and then co-cultured with a human alveolar basal epithelial cell line (A549) or endothelial cell line (HUVEC) for 24 h. Cell viability was determined (viability dye^+^cells) by flow cytometry. We found that SARS-CoV-2–activated neutrophils reduced the viability of A549 and HUVEC cells compared with non-activated neutrophils. Importantly, disulfiram treatment reduced the cell death induced by SARS-CoV-2–activated neutrophils (Fig. 5A–D and Additional file 1: Fig. S7). These results indicate that the GSDMD inhibition can prevent tissue damage mediated by SARS-CoV-2–induced NETs.

### Disulfiram prevents NETs release and organ dysfunction in a COVID-19 experimental model

**Fig. 6:**
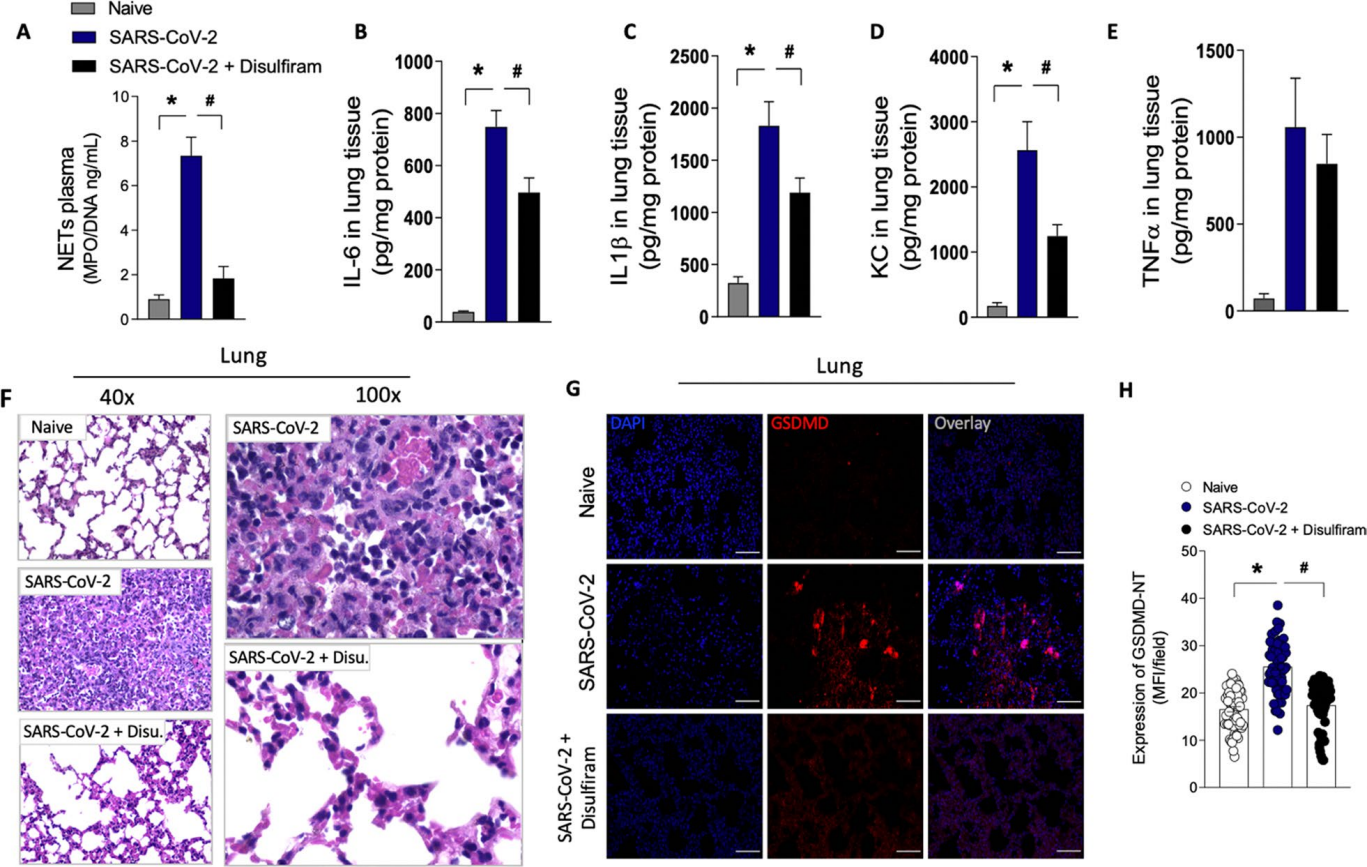
Pharmacological inhibition of GSDMD prevents NET release, lung inflammation, and organ damage in a mouse model of COVID-19**.** ACE-2 humanized mice were infected with SARS-CoV-2, and after 24 h, mice were treated with disulfiram (50 mg/kg, i.p. 1 × per day, during 5 days) or vehicle. **A** The MPO/DNA-NET concentration in the plasma was determined 5 days post-SARS- CoV-2 infection. **B–E** The levels of inflammatory cytokines (IL-6, IL-1β, CXCL-1/KC, and TNF-α) in lung tissue were measured by ELISA 5 days post-SARS-CoV-2 infection. **F** Representative images of the histological staining of the lung sections performed 5 days post-SARS-CoV-2 infection are shown at 200× magnification and 400× magnification. **G** Representative confocal analysis of GSDMD-NT and NETs in the lung tissue sample. Immunostaining for DNA (DAPI, blue) and the GSDMD cleaved fraction (GSDMD-NT, red) are shown. The scale bar indicates 50 μm at 630 × magnification. **H** GSDMD-NT expression was quantified by MFI per field. The data are expressed as means ± SEM (*or ^#^ *p* < 0.05; one-way ANOVA followed by Tukey’s test in **A**–**E** and **H**). The data are representative of at least two independent experiments, each including 5–7 animals per group

To investigate the importance of GSDMD-dependent NETosis to COVID-19 immunopathology, we infected K18 hACE2 transgenic mice with SARS-CoV-2 and treated them with disulfiram. According to Oladunni et al. 2020 [26], mice submitted to SARS-CoV infection showed a dramatic reduction in the overall survival curves. In a preliminary experiment, we confirmed these results. Therefore, we perform euthanasia on day 5 after the virus inoculation to obtain tissue and blood samples for analysis. Corroborating with the *in vitro* findings, we found that disulfiram treatment did not reduce the viral load after SARS-CoV-2 infection (Additional file 1: Fig. S8). However, the treatment reduced the circulating levels of NETs after SARS-CoV-2 infection compared to the group treated with vehicle (Fig. 6A). Additionally, the levels of inflammatory cytokines IL-6, IL-1β, and CXCL1/KC, but not TNF-α, in lung tissue were also mitigated by treatment with disulfiram (Fig. 6, B-E). Histological analysis of the lung tissue from SARS-CoV-2 infected mice showed a septal thickening by intense neutrophil infiltration with alveolar-capillary barrier damage. At higher magnification, we also observed the parenchymal lung remodeling with architectural distortion and an intense inflammatory cells infiltration with damage of pneumocytes and endothelial cells of the alveolar septa. Importantly, the treatment of infected mice with disulfiram reduced these inflammatory events, avoiding alveolar septal thickening and preserving the tissue histoarchitecture (Fig. 6F). Furthermore, the confocal microscopy analysis of lung tissues showed that disulfiram treatment markedly reduced the GSDMD-NT expression after SARS-CoV-2 infection (Fig. 6G and H). Although lung injuries are a hallmark of COVID-19, evidence has shown that other organs are also affected [33, 34]. Thus, we analyze the protective effect of disulfiram in other tissues. We observed that the heart of animals infected with SARS-CoV-2 showed diffuse and sparse cardiac interstitial inflammatory infiltrate, with perivascular accentuation (Additional file 1: Fig. S9A). In the kidney tissue, the SARS-CoV-2 infection-induced ischemic tubulointerstitial nephritis, mimicking acute tubular necrosis, which was associated with cell glomerulitis (Additional file 1: Fig. S9B). In the liver of infected mice, central-portal necroinflammatory hepatitis and spillover with piecemeal necrosis were found (Additional file 1: Fig. S9C). The treatment with disulfiram promoted the preservation of tissue architecture, reduced the inflammatory infiltrate, and attenuated tissue damage. Collectively, these findings demonstrate that pharmacological inhibition of GSDMD with disulfiram prevents NETs release and organ dysfunction and can be used to improve the COVID-19 treatment.

## Discussion

We and others have identified NETs as potential drivers of COVID-19 severity [6–9]. The massive release of NETs is associated with systemic inflammation, organ damage, and thrombosis that is found in severe cases of COVID-19 [6–9]. Remarkably, we reported that SARS-CoV-2 can directly stimulate human neutrophils to release NETs [7]. However, how SARS-CoV-2 leads to the release of NETs is still unclear. The present study reveals that the virus that causes COVID-19 directly triggers NET release by a GSDMD pathway-dependent manner. We found that GSDMD is expressed in lung tissue of patients with severe COVID-19 in association with NETs structures. The release of NETs by neutrophils infected with SARS-CoV-2 or isolated from patients with severe COVID-19 was inhibited with GSDMD inhibitor, disulfiram. Importantly, we observed an association between the level of cleaved GSDMD and severity of COVID-19. Moreover, in a mouse model of COVID-19, the treatment with disulfiram abrogated NETs release and reduced organ damage.

The neutrophil death by NETose depends on two basic events, the synthesis of NETs and their release by the neutrophils. The synthesis is mediated by ROS production, calcium mobilization, followed by PAD-4 or neutrophil elastase activation. Then, these enzymes transmigrate to the nucleus, where they mediate the citrullination or cleavage of histones promoting chromatin decondensation, respectively [5, 35]. Recently, it was demonstrated that the release of NETs depends on the formation of pores on nuclear and plasma membranes, which are mediated by cleaved GSDMD [10, 11]. Several pieces of evidences also have shown that, regardless of the stimuli, during the ongoing of several diseases, the NETosis processes require a convergence of signaling pathways involved in the synthesis and release of NETs [5, 8, 35]. These pathways could be directed activated by DAMPs and PAMPs via TLRs or indirectly, as it is observed via platelet activation [5, 35, 36]. In the context of COVID-19, we and others described the participation of PAD4 and ROS in the process of NETosis induced by SARS-CoV-2 [7, 37]. Nonetheless, the mechanisms involved in the release of NETs are not well characterized. In the present study, we demonstrated that activation of GSDMD via caspase1/4 is required for the release of NETs and this event depends on virus entry into the cell via ACE2 or TMPRSS2 and also de viral replication indicating that intracellular mechanisms of virus recognition may be involved.

A possible link connecting the activation of GSDMD by SARS-CoV-2 infection and replication relies on mechanisms triggered by intracellular RNA sensors. Previous reports indicate that the intracellular RNA sensor RIG-I is involved in the recognition of SARS-CoV-2 [38]. The RIG-I activation assembles caspase-1-activating inflammasome complexes which mediate GSDMD cleavage [31, 39]. In this context, we observed an increase in RIG-1 expression in neutrophils from COVID-19 patients. Thus, the possibility of RIG-I being involved in GSDMD activation via caspases/NLRP3 during SARS-CoV-2 infection deserves future investigation. However, it is important to mention that GSDMD is the final signal for the release of NETs induced by SARS-CoV-2. Similarly, we recently demonstrated that during bacterial sepsis [14] and neutrophils stimulated with bacteria (*Streptococcus pneumoniae or Staphylococcus aureus*), as shown in the present study, the NET production is dependent on GSDMD activation. Therefore, we propose that GSDMD is a target to ameliorate COVID-19 therapy.

Disulfiram is a drug approved for the treatment of alcohol dependence for its inhibitory effect on aldehyde dehydrogenase (ALDH) [15, 40]. However, a study showed that disulfiram at nanomolar concentration covalently binds and modifies human/mouse Cys191/Cys192 in GSDMD inhibiting its pore-forming function [16]. Furthermore, we recently demonstrated that inhibition of GSDMD by disulfiram prevents neutrophil death by NETosis [14]. Considering this finding, we tested the effect of disulfiram on neutrophils infected by SARS-CoV-2 and in the COVID-19 experimental model. *In vitro* we observed that disulfiram inhibited NETs production induced by SARS-CoV-2 through GSDMD inhibition, without impacting viral replication. Furthermore, we analyze if disulfiram might prevent the cell damage induced by NETs. For this, we used classical models described in the literature to determine cytotoxicity of NETs, Human lung epithelial cell line (A549 cell line) and human umbilical vein endothelial cells line (HUVEC cells) (41–44). We demonstrated that the release of the NETs involved in this cytotoxicity is dependent on GSDMD activation. Then, we use K18hACE2 mice with humanized ACE2 to investigate the potential therapeutic effect of disulfiram. The K18 hACE2 transgenic mice was succumbed to SARS-CoV-2 infection by day 6, and with virus detected in lung airway epithelium and brain [26], we reproduced these data in our conditions at the dose of 2 × 10^4^ PFU. Considering that we perform daily treatment starting 24h after the virus inoculation, and on day 5, mice were euthanized to collect samples. As demonstrated *in vitro,* disulfiram treatment did not reduce the viral load after SARS-CoV-2 infection, but the therapy was sufficient to reduce NETs release, cytokine storm, and attenuate tissues damages in several organs. Similarly, we observed that the inhibition of GSDMD by disulfiram in the sepsis model efficiently abrogates NETosis, systemic inflammation, and vital organs dysfunction, improving mice survival [14]. Of note, the course of the disease in the mouse model is different when compared to humans, in which the symptoms are observed on 5–6 days after the infection and maintained for around 14 days [45].

Although the effect of GSDMD inhibition by disulfiram may be associated with the reduction of NETs, we do not exclude its effect in other cells, such as macrophages, blocking the release of inflammatory cytokines, as observed in the lung tissue of infected mice treated with disulfiram.

## Conclusions

Taken together, our findings demonstrate that GSDMD plays a critical role in the generation of NETs and organ damage induced by SARS-CoV-2 infection. Therefore, the pharmacological inhibition of GSDMD, as with disulfiram, represents a potential strategy to improve the treatment of COVID-19.

## Supplementary Information

**Additional file 1**. Supplemental data including GSDMD expression in airway fluid, antiviral effect of disulfiram, viral replication in neutrophils, RIG-I expression in neutrophils, flow cytometry gating strategy as well as histopathology of mice organs.

## Data Availability

The datasets used and/or analyzed during the current study are available from the corresponding author on reasonable request.

## References

[1] Brinkmann V, Reichard U, Goosmann C, Fauler B, Uhlemann Y, Weiss DS, Weinrauch Y, Zychlinsky A (2004). Neutrophil extracellular traps kill bacteria. Science (New York, N.Y.).

[2] Knight JS, Luo W, O'Dell AA, Yalavarthi S, Zhao W, Subramanian V, Guo C, Grenn RC, Thompson PR, Eitzman DT, Kaplan MJ (2014). Peptidylarginine deiminase inhibition reduces vascular damage and modulates innate immune responses in murine models of atherosclerosis. Circ Res.

[3] Carmona-Rivera C, Carlucci PM, Goel RR, James E, Brooks SR, Rims C, Hoffmann V, Fox DA, Buckner JH, Kaplan MJ (2020). Neutrophil extracellular traps mediate articular cartilage damage and enhance cartilage component immunogenicity in rheumatoid arthritis. JCI Insight.

[4] Czaikoski PG, Mota JM, Nascimento DC, Sônego F, Castanheira FV, Melo PH, Scortegagna GT, Silva RL, Barroso-Sousa R, Souto FO, Pazin-Filho A, Figueiredo F, Alves-Filho JC, Cunha FQ (2016). Neutrophil extracellular traps induce organ damage during experimental and clinical sepsis. PLoS ONE.

[5] Papayannopoulos V (2018). Neutrophil extracellular traps in immunity and disease. Nat Rev Immunol.

[6] Leppkes M, Knopf J, Naschberger E, Lindemann A, Singh J, Herrmann I, Stürzl M, Staats L, Mahajan A, Schauer C, Kremer AN, Völkl S, Amann K, Evert K, Falkeis C, Wehrfritz A, Rieker RJ, Hartmann A, Kremer AE, Neurath MF (2020). Vascular occlusion by neutrophil extracellular traps in COVID-19. EBioMedicine.

[7] Veras FP, Pontelli MC, Silva CM, Toller-Kawahisa JE, de Lima M, Nascimento DC, Schneider AH, Caetité D, Tavares LA, Paiva IM, Rosales R, Colón D, Martins R, Castro IA, Almeida GM, Lopes M, Benatti MN, Bonjorno LP, Giannini MC, Luppino-Assad R (2020). SARS-CoV-2-triggered neutrophil extracellular traps mediate COVID-19 pathology. J Exp Med.

[8] Ackermann M, Anders HJ, Bilyy R, Bowlin GL, Daniel C, De Lorenzo R, Egeblad M, Henneck T, Hidalgo A, Hoffmann M, Hohberger B, Kanthi Y, Kaplan MJ, Knight JS, Knopf J, Kolaczkowska E, Kubes P, Leppkes M, Mahajan A, Manfredi AA (2021). Patients with COVID-19: in the dark-NETs of neutrophils. Cell Death Differ.

[9] Radermecker C, Detrembleur N, Guiot J, Cavalier E, Henket M, d'Emal C, Vanwinge C, Cataldo D, Oury C, Delvenne P, Marichal T (2020). Neutrophil extracellular traps infiltrate the lung airway, interstitial, and vascular compartments in severe COVID-19. J Exp Med.

[10] Sollberger G, Choidas A, Burn GL, Habenberger P, Di Lucrezia R, Kordes S, Menninger S, Eickhoff J, Nussbaumer P, Klebl B, Krüger R, Herzig A, Zychlinsky A (2018). Gasdermin D plays a vital role in the generation of neutrophil extracellular traps. Sci Immunol.

[11] Chen KW, Monteleone M, Boucher D, Sollberger G, Ramnath D, Condon ND, von Pein JB, Broz P, Sweet MJ, Schroder K (2018). Noncanonical inflammasome signaling elicits gasdermin D-dependent neutrophil extracellular traps. Sci Immunol.

[12] Kambara H, Liu F, Zhang X, Liu P, Bajrami B, Teng Y, Zhao L, Zhou S, Yu H, Zhou W, Silberstein LE, Cheng T, Han M, Xu Y, Luo HR (2018). Gasdermin D exerts anti-inflammatory effects by promoting neutrophil death. Cell Rep.

[13] Broz P, Pelegrín P, Shao F (2020). The gasdermins, a protein family executing cell death and inflammation. Nat Rev Immunol.

[14] Silva C, Wanderley C, Veras FP, Sonego F, Nascimento DC, Gonçalves AV, Martins TV, Cólon DF, Borges VF, Brauer VS, Damasceno L, Silva KP, Toller-Kawahisa JE, Batah SS, Souza A, Monteiro VS, Oliveira A, Donate PB, Zoppi D, Borges MC (2021). Gasdermin D inhibition prevents multiple organ dysfunction during sepsis by blocking NET formation. Blood.

[15] Koppaka V, Thompson DC, Chen Y, Ellermann M, Nicolaou KC, Juvonen RO, Petersen D, Deitrich RA, Hurley TD, Vasiliou V (2012). Aldehyde dehydrogenase inhibitors: a comprehensive review of the pharmacology, mechanism of action, substrate specificity, and clinical application. Pharmacol Rev.

[16] Hu JJ, Liu X, Xia S, Zhang Z, Zhang Y, Zhao J, Ruan J, Luo X, Lou X, Bai Y, Wang J, Hollingsworth LR, Magupalli VG, Zhao L, Luo HR, Kim J, Lieberman J, Wu H (2020). FDA-approved disulfiram inhibits pyroptosis by blocking gasdermin D pore formation. Nat Immunol.

[17] Fillmore N, Bell S, Shen C, Nguyen V, La J, Dubreuil M, Strymish J, Brophy M, Mehta G, Wu H, Lieberman J, Do N, Sander C (2021). Disulfiram use is associated with lower risk of COVID-19: a retrospective cohort study. PLoS ONE.

[18] Liao M, Liu Y, Yuan J, Wen Y, Xu G, Zhao J, Cheng L, Li J, Wang X, Wang F, Liu L, Amit I, Zhang S, Zhang Z (2020). Single-cell landscape of bronchoalveolar immune cells in patients with COVID-19. Nat Med.

[19] Hao Y, Hao S, Andersen-Nissen E, Mauck WM, Zheng S, Butler A, Lee MJ, Wilk AJ, Darby C, Zager M, Hoffman P, Stoeckius M, Papalexi E, Mimitou EP, Jain J, Srivastava A, Stuart T, Fleming LM, Yeung B, Rogers AJ (2021). Integrated analysis of multimodal single-cell data. Cell.

[20] Lex A, Gehlenborg N, Strobelt H, Vuillemot R, Pfister H (2014). UpSet: visualization of intersecting sets. IEEE Trans Visual Comput Graphics.

[21] Lopes MI, Bonjorno LP, Giannini MC, Amaral NB, Menezes PI, Dib SM, Gigante SL, Benatti MN, Rezek UC, Emrich-Filho LL, Sousa B, Almeida S, Luppino Assad R, Veras FP, Schneider A, Rodrigues TS, Leiria L, Cunha LD, Alves-Filho JC, Cunha TM (2021). Beneficial effects of colchicine for moderate to severe COVID-19: a randomised, double-blinded, placebo-controlled clinical trial. RMD Open.

[22] Wu Z, McGoogan JM (2020). Characteristics of and important lessons from the coronavirus disease 2019 (COVID-19) outbreak in China: Summary of a report of 72 314 cases from the chinese center for disease control and prevention. JAMA.

[23] Jin YH, Cai L, Cheng ZS, Cheng H, Deng T, Fan YP, Fang C, Huang D, Huang LQ, Huang Q, Han Y, Hu B, Hu F, Li BH, Li YR, Liang K, Lin LK, Luo LS, Ma J, Ma LL (2020). A rapid advice guideline for the diagnosis and treatment of 2019 novel coronavirus (2019-nCoV) infected pneumonia (standard version). Mil Med Res.

[24] Duarte-Neto AN, Monteiro R, Johnsson J, Cunha M, Pour SZ, Saraiva AC, Ho YL, da Silva L, Mauad T, Zanotto P, Saldiva P, de Oliveira I, Dolhnikoff M (2019). Ultrasound-guided minimally invasive autopsy as a tool for rapid post-mortem diagnosis in the 2018 Sao Paulo yellow fever epidemic: correlation with conventional autopsy. PLoS Negl Trop Dis.

[25] McCray PB, Pewe L, Wohlford-Lenane C, Hickey M, Manzel L, Shi L, Netland J, Jia HP, Halabi C, Sigmund CD, Meyerholz DK, Kirby P, Look DC, Perlman S (2007). Lethal infection of K18-hACE2 mice infected with severe acute respiratory syndrome coronavirus. J Virol.

[26] Oladunni FS, Park JG, Pino PA, Gonzalez O, Akhter A, Allué-Guardia A, Olmo-Fontánez A, Gautam S, Garcia-Vilanova A, Ye C, Chiem K, Headley C, Dwivedi V, Parodi LM, Alfson KJ, Staples HM, Schami A, Garcia JI, Whigham A, Platt RN (2020). Lethality of SARS-CoV-2 infection in K18 human angiotensin-converting enzyme 2 transgenic mice. Nat Commun.

[27] Bao L, Deng W, Huang B, Gao H, Liu J, Ren L, Wei Q, Yu P, Xu Y, Qi F, Qu Y, Li F, Lv Q, Wang W, Xue J, Gong S, Liu M, Wang G, Wang S, Song Z (2020). The pathogenicity of SARS-CoV-2 in hACE2 transgenic mice. Nature.

[28] Hoffmann M, Kleine-Weber H, Schroeder S, Krüger N, Herrler T, Erichsen S, Schiergens TS, Herrler G, Wu NH, Nitsche A, Müller MA, Drosten C, Pöhlmann S (2020). SARS-CoV-2 Cell entry depends on ACE2 and TMPRSS2 and is blocked by a clinically proven protease inhibitor. Cell.

[29] Clososki GC, Soldi RA, Guaratini T, Martins RB, Costa CS, Arruda E, Lopes NP (2020). Tenofovir disoproxil fumarate: new chemical developments and encouraging. J Braz Chem Soc.

[30] Shi J, Zhao Y, Wang K, Shi X, Wang Y, Huang H, Zhuang Y, Cai T, Wang F, Shao F (2015). Cleavage of GSDMD by inflammatory caspases determines pyroptotic cell death. Nature.

[31] Elion DL, Cook RS (2018). Harnessing RIG-I and intrinsic immunity in the tumor microenvironment for therapeutic cancer treatment. Oncotarget.

[32] Zeng H, Ma Y, Zhou Z, Liu W, Huang P, Jiang M, Liu Q, Chen P, Luo H, Chen Y (2021). Spectrum and clinical characteristics of symptomatic and asymptomatic coronavirus disease 2019 (COVID-19) with and without Pneumonia. Front Med.

[33] Zhang C, Shi L, Wang FS (2020). Liver injury in COVID-19: management and challenges. Lancet Gastroenterol Hepatol.

[34] Shi S, Qin M, Shen B, Cai Y, Liu T, Yang F, Gong W, Liu X, Liang J, Zhao Q, Huang H, Yang B, Huang C (2020). Association of cardiac injury with mortality in hospitalized patients with COVID-19 in Wuhan, China. JAMA Cardiol.

[35] Jorch SK, Kubes P (2017). An emerging role for neutrophil extracellular traps in noninfectious disease. Nat Med.

[36] Funchal GA, Jaeger N, Czepielewski RS, Machado MS, Muraro SP, Stein RT, Bonorino CB, Porto BN (2015). Respiratory syncytial virus fusion protein promotes TLR-4-dependent neutrophil extracellular trap formation by human neutrophils. PLoS ONE.

[37] Arcanjo A, Logullo J, Menezes C, de Souza Carvalho Giangiarulo TC, Dos Reis MC, de Castro G, da Silva Fontes Y, Todeschini AR, Freire-de-Lima L, Decoté-Ricardo D, Ferreira-Pereira A, Freire-de-Lima CG, Barroso S, Takiya C, Conceição-Silva F, Savino W, Morrot A (2020). The emerging role of neutrophil extracellular traps in severe acute respiratory syndrome coronavirus 2 (COVID-19). Sci Rep.

[38] Yamada T, Sato S, Sotoyama Y, Orba Y, Sawa H, Yamauchi H, Sasaki M, Takaoka A (2021). RIG-I triggers a signaling-abortive anti-SARS-CoV-2 defense in human lung cells. Nat Immunol.

[39] Rintahaka J, Wiik D, Kovanen PE, Alenius H, Matikainen S (2008). Cytosolic antiviral RNA recognition pathway activates caspases 1 and 3. J Immunol (Baltimore, Md.: 1950).

[40] Wright C, Moore RD (1990). Disulfiram treatment of alcoholism. Am J Med.

[41] Villanueva E, Yalavarthi S, Berthier CC, Hodgin JB, Khandpur R, Lin AM, Rubin CJ, Zhao W, Olsen SH, Klinker M, Shealy D, Denny MF, Plumas J, Chaperot L, Kretzler M, Bruce AT, Kaplan MJ (2011). Netting neutrophils induce endothelial damage, infiltrate tissues, and expose immunostimulatory molecules in systemic lupus erythematosus. J immunol (Baltimore, Md. : 1950).

[42] Muraro SP, De Souza GF, Gallo SW, Da Silva BK, De Oliveira SD, Vinolo M, Saraiva EM, Porto BN (2018). Respiratory Syncytial Virus induces the classical ROS-dependent NETosis through PAD-4 and necroptosis pathways activation. Sci Rep.

[43] Lv D, Xu Y, Cheng H, Ke Y, Zhang X, Ying K (2020). A novel cell-based assay for dynamically detecting neutrophil extracellular traps-induced lung epithelial injuries. Exp Cell Res.

[44] Lee HW, Nizet V, An JN, Lee HS, Song YR, Kim SG, Kim JK (2021). Uremic serum damages endothelium by provoking excessive neutrophil extracellular trap formation. Sci Rep.

[45] Zhou F, Yu T, Du R, Fan G, Liu Y, Liu Z, Xiang J, Wang Y, Song B, Gu X, Guan L, Wei Y, Li H, Wu X, Xu J, Tu S, Zhang Y, Chen H, Cao B (2020). Clinical course and risk factors for mortality of adult inpatients with COVID-19 in Wuhan, China: a retrospective cohort study. Lancet (London, England).

