# Supplementary material for: Gasdermin-D activation by SARS-CoV-2 triggers NET and mediate COVID-19 immunopathology
Source: Crit Care. 2022 Jul 7;26:206. doi: 10.1186/s13054-022-04062-5 (PMC9261892; doi:10.1186/s13054-022-04062-5)
Supplement: Supplementary file 1 — Additional file 1. Supplemental data including GSDMD expression in airway fluid, antiviral effect of disulfiram, viral replication in neutrophils, RIG-I expression in neutrophils, flow cytometry gating strategy as well as histopathology of mice organs. [file 13054_2022_4062_MOESM1_ESM.docx]

**Supplementary Figures**

**Supplementary figure 1. GSDMD expression is associated with NETs in airway fluid from COVID-19 severe patients. (A)** NET quantification by picogreen assay with MPO-DNA in the airway fluid from COVID-19 patients (n=11) and in saline-induced airway fluids from healthy control (n=8). **(B)** Representative confocal analysis of GSDMD and NETs from the airway fluid of COVID-19 severe patients. Cells were stained for DNA (DAPI, blue), MPO (green), and GSDMD-NT (red). The scale bar indicates 50 μm. 4× digital zoom was performed in the inset white square. The data are expressed as mean ± SEM (*p<0.05; t-test in A).


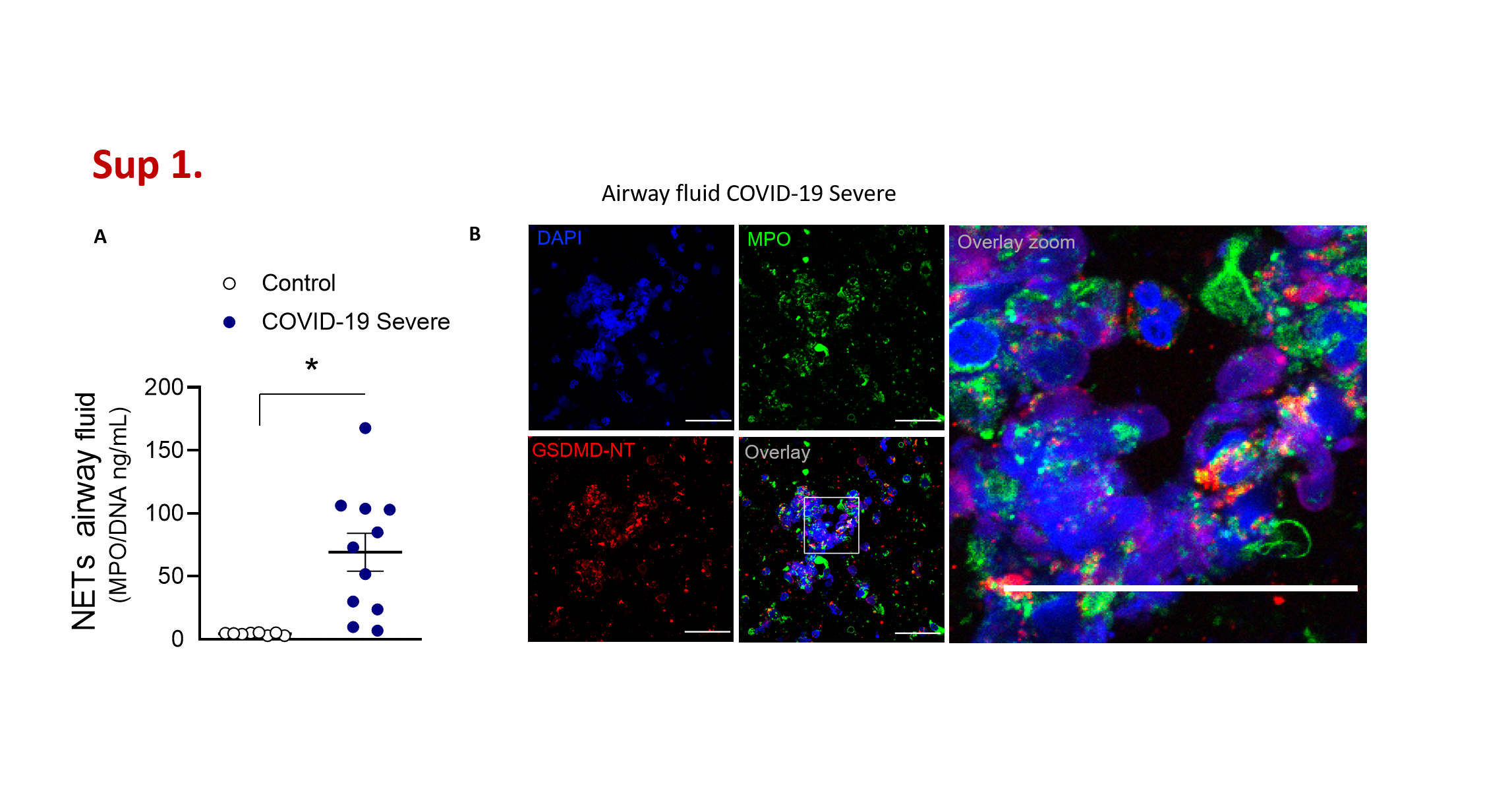

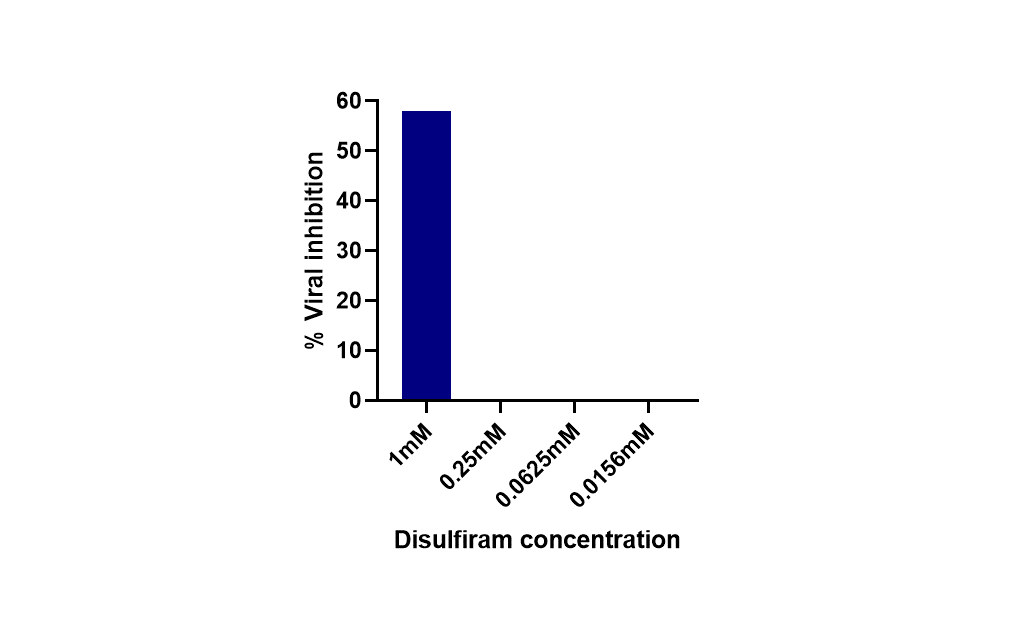


**Supplementary figure 2. Evaluation of the antiviral effect of disulfiram against SARS-CoV-2.** Vero E6 cells were treated with disulfiram (using a 4-fold serial dilution) and then incubated 1 hour at 37ºC with approximately 90 PFU (Plaque Forming Units) of SARS-CoV-2. Four days after infection, viral release to the media was measured by the plaque-forming unit (PFU) assay.


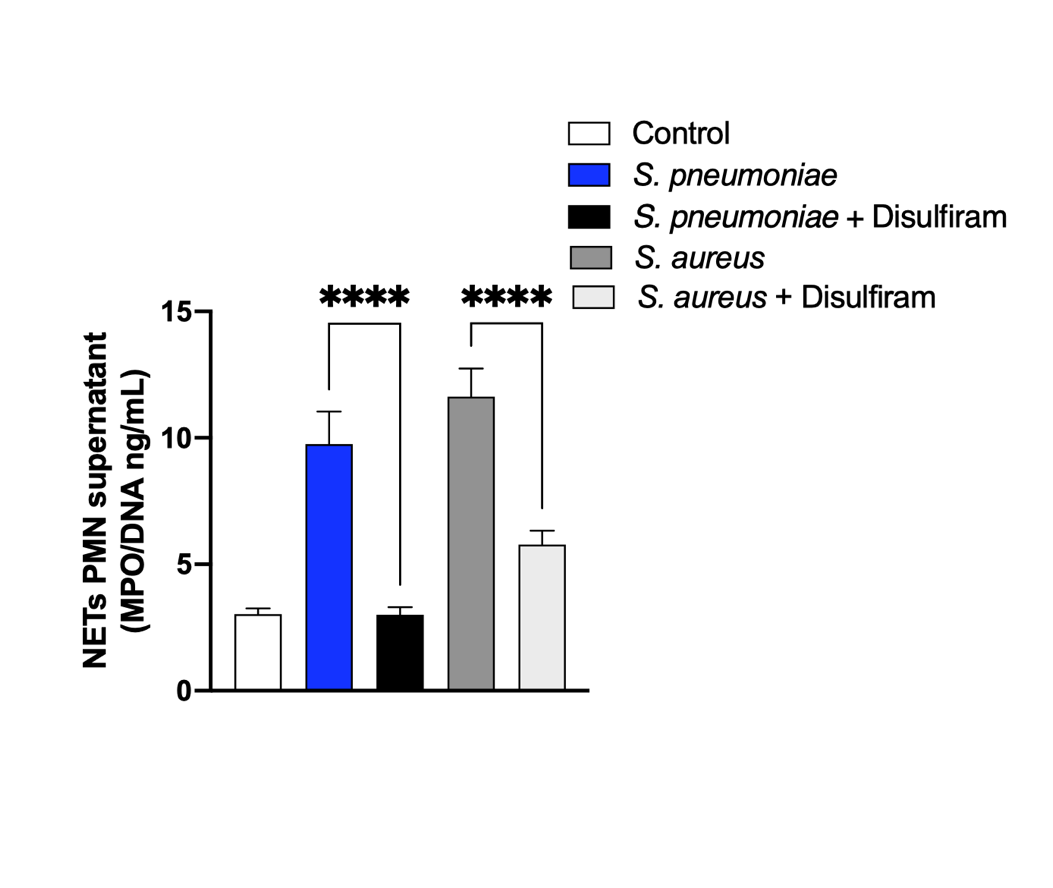


**Supplementary figure 3. Disulfiram treatment prevents NETosis induced by bacteria.** Human neutrophils were isolated from healthy volunteers. Cells were treated with disulfiram (30 µM) and after 1 h cells were incubated with medium (control), *Streptococcus pneumoniae (*1:1*),* or *Staphylococcus aureus (*3:1*)* for 4 h at 37°C. The concentrations of MPO/DNA-NETs in the supernatants were determined using the picogreen test. The data are expressed as mean ± SEM (*p<0.05 one-way ANOVA followed by Tukey’s test).

**
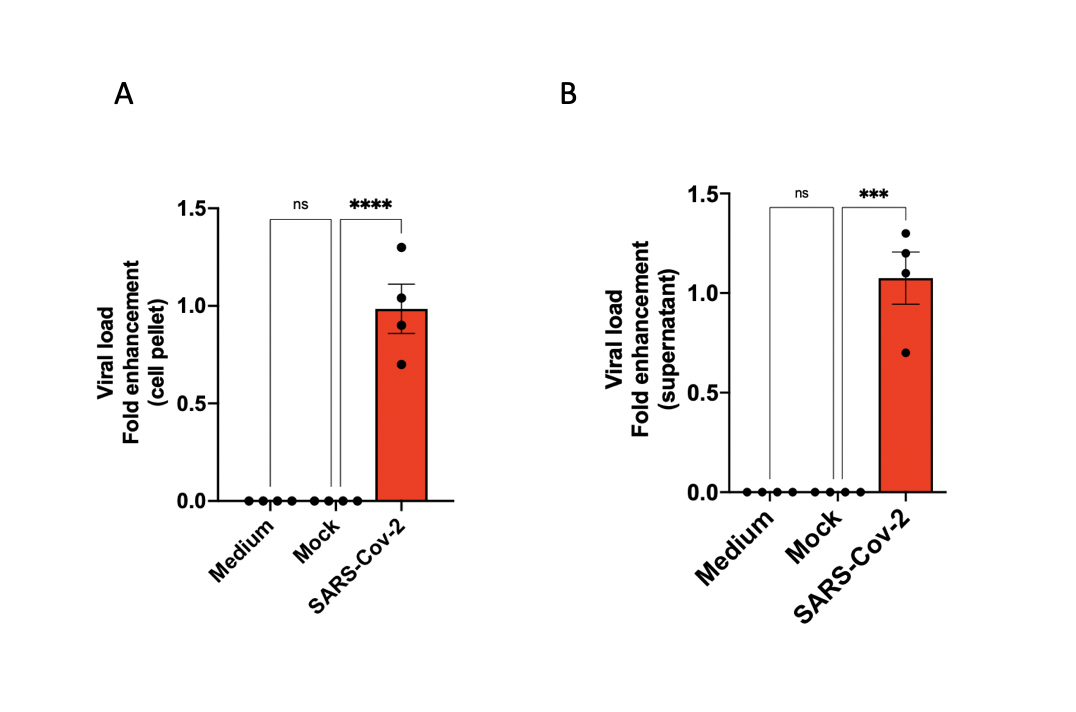
**

**Supplementary** **figure 4.**  **SARS-CoV-2 viral replication in human neutrophils.** Human neutrophils were isolated from healthy volunteers. Cells were incubated with medium, virus control (Mock), or SARS-CoV-2, for 4 h at 37°C. SARS-CoV-2 viral load detection in **(A)** neutrophil cell pellet and **(B)** in the supernatant by RT-PCR, 4 h after infection. Fold change relative to SARS-CoV-2 group was used. The data are expressed as mean ± SEM (*p<0.05; one-way ANOVA followed by Tukey’s test).


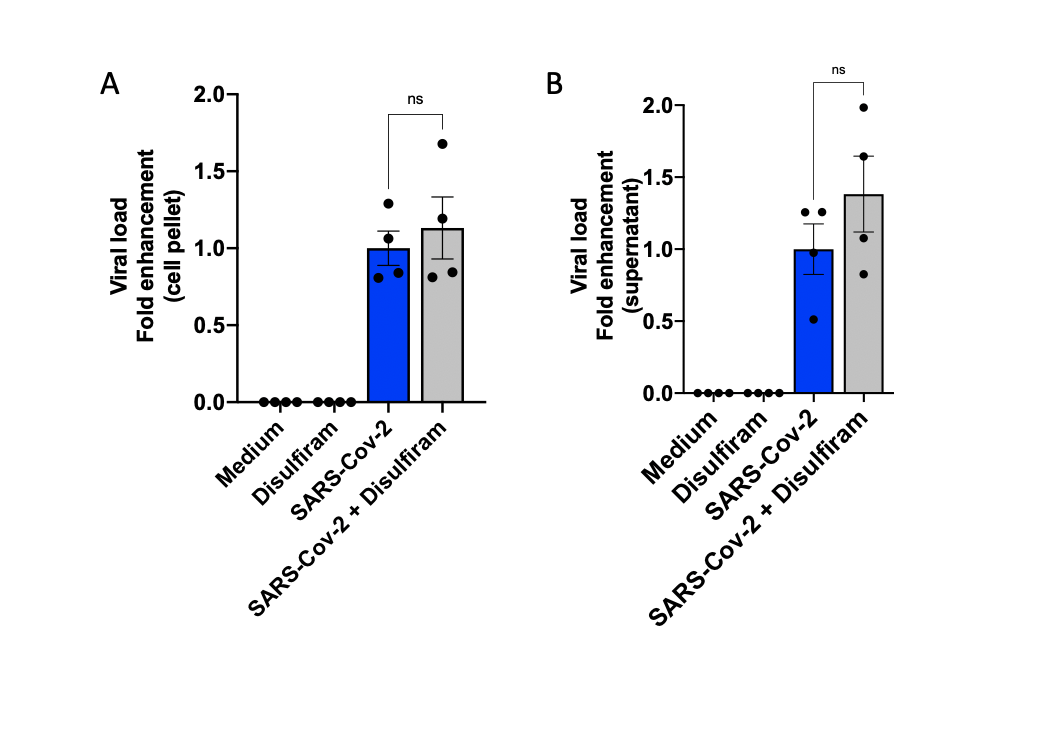


**Supplementary** **figure 5.**  **SARS-CoV-2 viral replication in human neutrophils is not affected by disulfiram.** Human neutrophils were isolated from healthy volunteers. Cells were treated with disulfiram (30 µM) and after 1 h the cells were incubated with medium, disulfiram, SARS-CoV-2, or SARS-CoV-2 + disulfiram for 4 h at 37°C. SARS-CoV-2 viral load detection in **(A)** neutrophil cell pellet and **(B)** in the supernatant by RT-PCR, 4 h after infection. Fold change relative to SARS-CoV-2 group was used. The data are expressed as mean ± SEM (*p<0.05; one-way ANOVA followed by Tukey’s test).


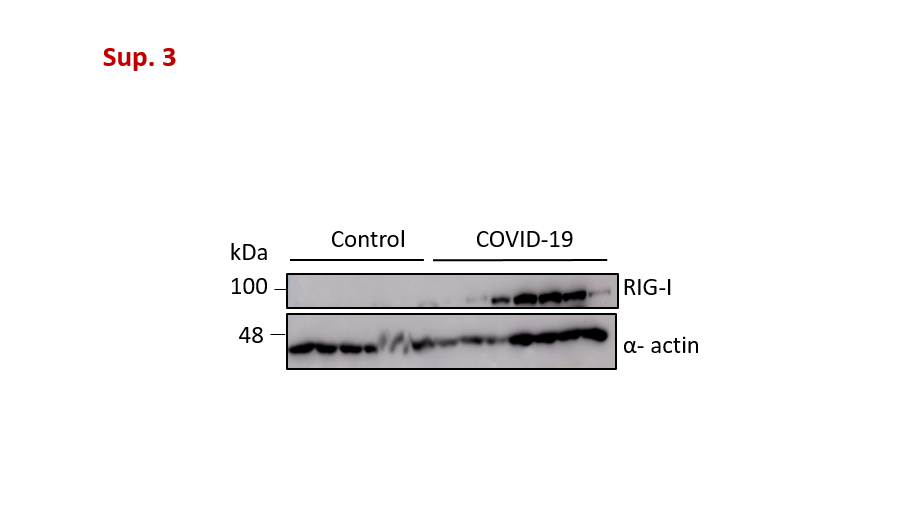
**Supplementary figure 6. RIG-I is highly expressed in neutrophils from COVID-19 patients.** Neutrophils were isolated from healthy controls (n=7) and COVID-19 patients (n=8). The neutrophil lysates were harvested for immunoblot analysis of RIG-1. The α-actin was used as a loading control.


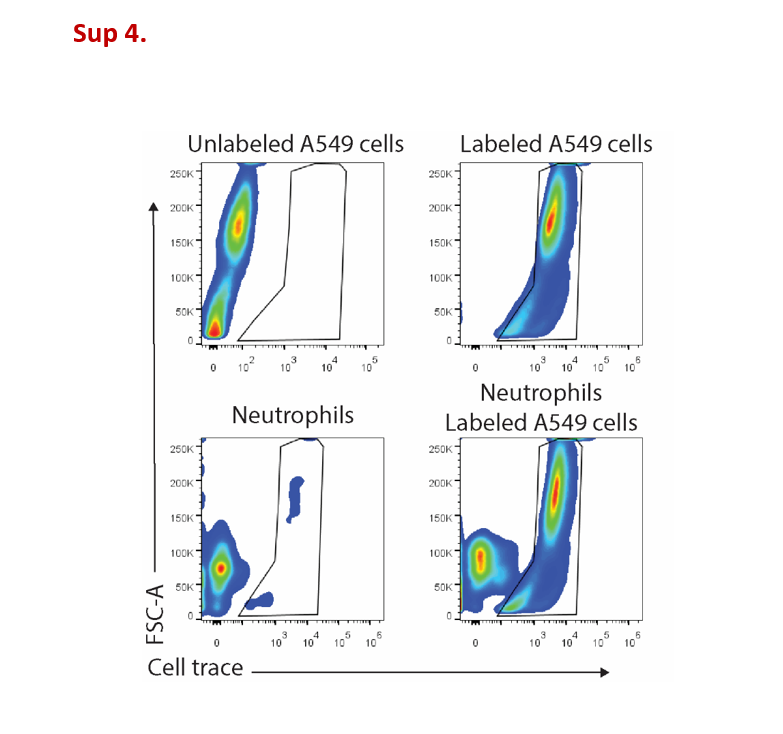


**Supplementary figure 7. Gating strategy for flow cytometry analysis.** Blood isolated neutrophils (10^6^ cells) from healthy donors, pretreated, or not, with disulfiram (30 µM) were incubated, or not, with SARS-CoV-2. After 1 h, these cells were washed twice and co-cultured with lung epithelial cells (A549, 2 × 10^5^ cells) or endothelial cells (HUVEC, 2 × 10^5^ cells) previously stained with viability dye for 24 h at 37°C. Gating strategy for flow cytometry analysis of A549 or HUVEC viability.

**
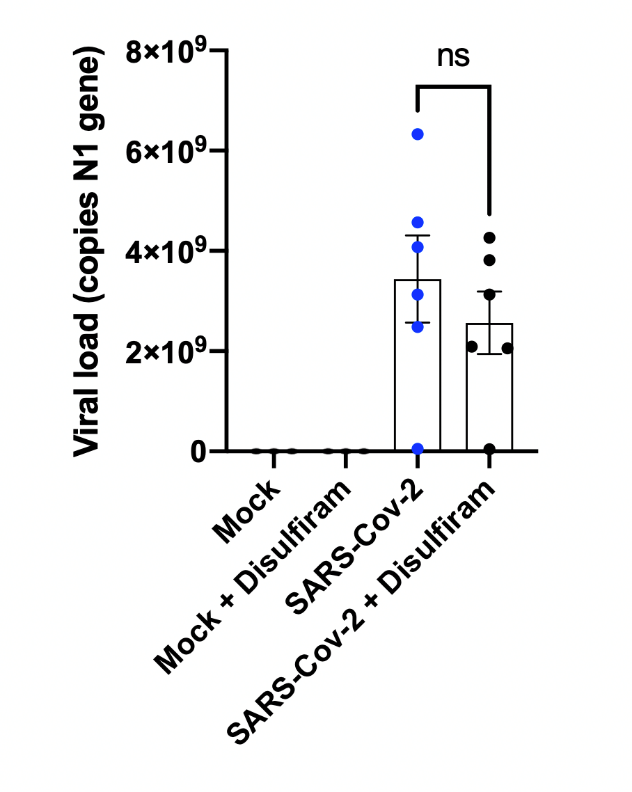
**

**Supplementary figure 8.** **Disulfiram does not alter SARS-CoV-2 replication in hACE2 mice.** ACE-2 humanized mice were infected with SARS-CoV-2 and after 24 h mice were treated with disulfiram (50 mg/kg, i.p. 1x per day, during 5 days) or vehicle. Viral load in the lung was collected from SARS-CoV-2–infected hACE2 mice on day 5. The data are expressed as mean ± SEM (*p<0.05; one-way ANOVA followed by Tukey’s test).


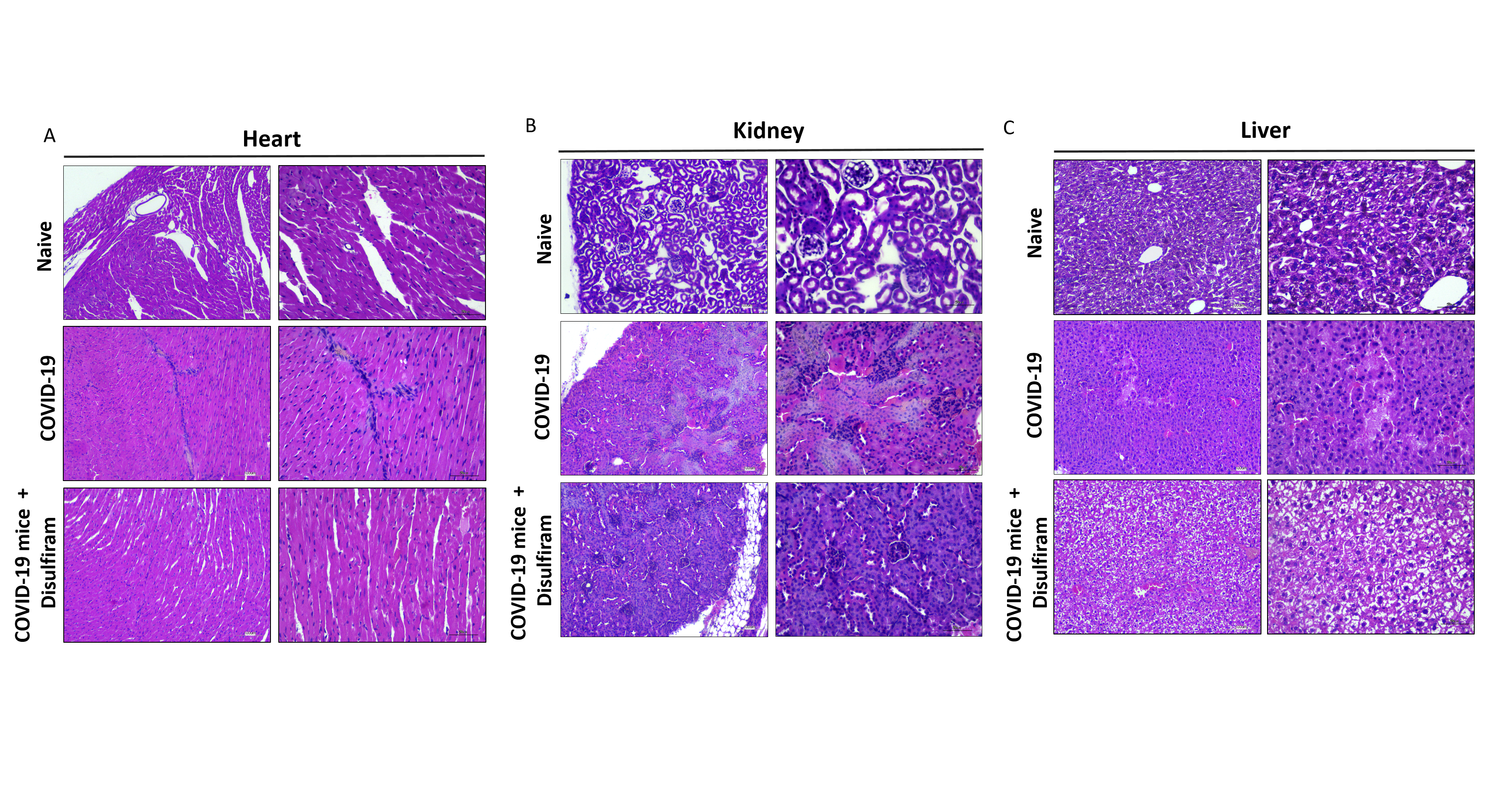


**Supplementary figure 9. GSDMD inhibition reduces organ damage in a COVID-19 mouse model.**  ACE-2 humanized mice were infected with SARS-CoV-2 (2x10^4^) and after 24 h we treated mice with disulfiram (50 mg/kg, i.p. 1x per day, during 5 days) or vehicle. **(A)** Representative images of the histological staining of the **(A)** heart, **(B)** kidney, and **(C)** Liver sections performed 5 days post-SARS-CoV-2 infection are shown at 200× magnification and at 400× magnification. The data are representative of groups, each including 5-7 animals per group.
